# Message in a molecule

**DOI:** 10.1038/ncomms11374

**Published:** 2016-05-03

**Authors:** Tanmay Sarkar, Karuthapandi Selvakumar, Leila Motiei, David Margulies

**Affiliations:** 1 Department of Organic Chemistry, Weizmann Institute of Science, Rehovot 7610001, Israel

## Abstract

Since ancient times, steganography, the art of concealing information, has largely relied on secret inks as a tool for hiding messages. However, as the methods for detecting these inks improved, the use of simple and accessible chemicals as a means to secure communication was practically abolished. Here, we describe a method that enables one to conceal multiple different messages within the emission spectra of a unimolecular fluorescent sensor. Similar to secret inks, this molecular-scale messaging sensor (m-SMS) can be hidden on regular paper and the messages can be encoded or decoded within seconds using common chemicals, including commercial ingredients that can be obtained in grocery stores or pharmacies. Unlike with invisible inks, however, uncovering these messages by an unauthorized user is almost impossible because they are protected by three different defence mechanisms: steganography, cryptography and by entering a password, which are used to hide, encrypt or prevent access to the information, respectively.

## Abstract (web summary)

Although historically common chemicals were frequently used as secret inks, the ease of readout could not prevent unauthorized reading. Here, the authors report a multi-analyte sensor that can conceal and encrypt messages by responding to simple chemicals, demonstrating a chemical means to secure communication.

## Graphical abstract

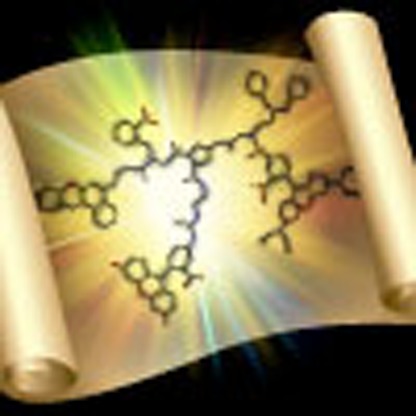

Nowadays, the use of invisible inks to write messages, which can be revealed only when exposed to heat, light or a chemical solution, is mostly associated with children's games. However, only a century ago exceptionally simple chemicals were frequently used in times of war for espionage purposes^1^,^2^. The main advantage of using these inks was their accessibility to field agents, which enabled straightforward writing and reading of confidential information^3^. However, one drawback of using this technology is the ease by which messages can be exposed, which has led, for example, to the capture of the ‘lemon juice spies' in World War I (WWI)^1^. A significant improvement in the ability to secure information by chemical means has been achieved with the development of molecular and biomolecular steganographic systems, in which specific chemical stimuli trigger the appearance of text and images. These data can be created by various sources, such as fluorescent materials^4^,^5^,^6^,^7^,^8^,^9^,^10^,^11^,^12^, bacteria^13^, antibodies^14^, photonic crystals^15^, NMR chemical shifts^16^ and molecular computing systems^17^,^18^,^19^,^20^. Another important advantage of using molecular steganography systems, namely, their small scale, has also been demonstrated by the ability to conceal messages within individual DNA strands^21^. Finally, advances in the area of molecular logic gates^22^,^23^,^24^,^25^,^26^ have resulted in alternative methods of securing information^22^,^27^,^28^ by using multi-analyte fluorescent molecular sensors that can produce ID-codes^29^ or can authorize password entries^30^,^31^,^32^,^33^,^34^,^35^,^36^,^37^,^38^,^39^,^40^,^41^.

Herein we present a different approach to molecular information protection, which relies on the ability of a molecular-scale messaging sensor (m-SMS) to convert randomly selected chemical signals into unpredictable emission patterns and, in doing so, communicate short, chemically encoded messages with maximal security. This sensor is the second member of the combinatorial fluorescent molecular sensor family, developed by our group^42^, which mimics the function of the olfactory system by integrating several nonspecific signalling receptors on a single molecular platform^43^. Unlike its predecessor^41^,^42^,^43^, however, or any other fluorescent probe that responds to several analytes^24^,^44^ or an analyte group^43^, m-SMS was designed to operate as a universal sensor that can discriminate among a vast number of distinct chemical species. We show that this property not only distinguishes m-SMS from other types of fluorescent molecular sensors, but also from other chemical security systems^4^,^5^,^6^,^7^,^8^,^9^,^10^,^11^,^12^,^13^,^14^,^15^,^16^,^17^,^18^,^19^,^20^,^21^,^22^,^27^,^28^,^29^,^30^,^31^,^32^,^33^,^34^,^35^,^36^,^37^,^38^,^39^,^40^,^41^ by enabling it to function as a molecular cipher device that can convert distinct chemical structures into unique encryption keys. In this way, the system can be used not only to hide the data (steganography), but also to encrypt and decrypt it (cryptography), as well as provide password protection when a higher level of security is needed. Because this system does not depend on using specific chemical inputs, unique instrumentations or complex experimental protocols, it is also very simple to operate. We show that m-SMS and/or the chemical ingredients can be concealed and delivered on plain letter paper and that the messages can be rapidly revealed using a low-cost, handheld spectrometer. This makes the m-SMS technology similar to the ancient technology of invisible inks in terms of simplicity, accessibility and the ease by which different messages can be concealed and exposed using common chemicals from various locations and in a short time.

## Results

### Design principles

**Figure 1:**
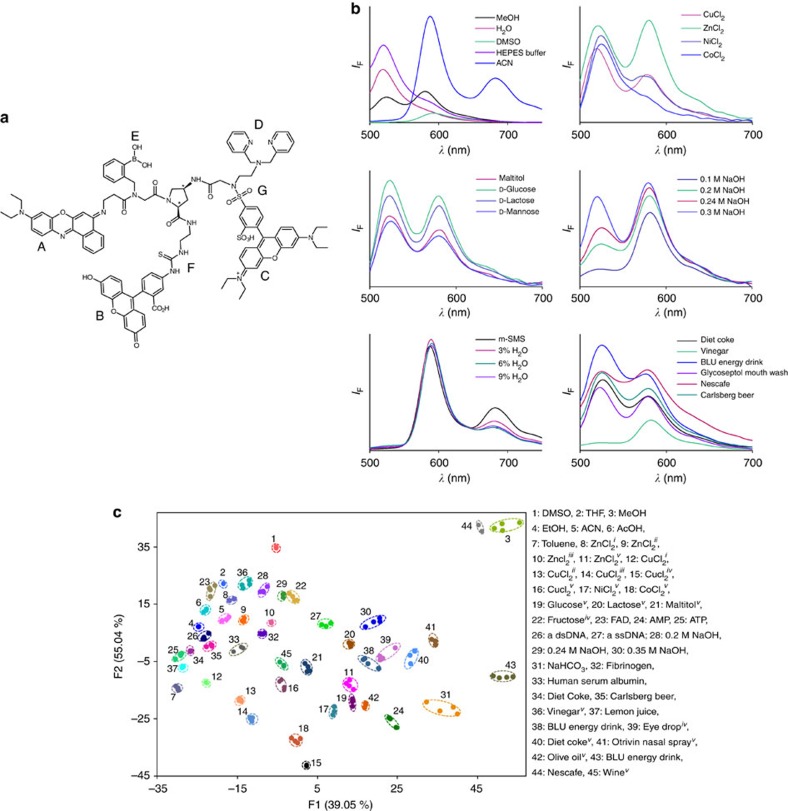
m-SMS operates as a universal sensor that can discriminate among multiple different analytes. (**a**) The structure of m-SMS integrates three fluorophores: solvatochromic nile blue (A), pH-sensitive fluorescein (B) and sulforhodamine B (C), as well as distinct recognition elements, such as dipicolylamine (D), boronic acid (E), thiourea (F) and sulfonamide (G). (**b**) Representative emission patterns generated by m-SMS in response to different analytes or conditions. The emission was recorded in different solvents (top left) and upon adding 2 μl of an aqueous solution of metal ions* (top right, 300 mΜ) and saccharides* (middle left, 13 mM) or by changing the pH** (middle right, 0.1–0.3 M NaOH), polarity*** (bottom left, 3–9% H_2_O) and upon adding commercial products* (bottom right). Initial conditions: m-SMS in *EtOH-AcOH (10 mM) and NaOH (11 mM), **EtOH-AcOH (10 mM) and ***acetonitrile (ACN). The concentration of m-SMS was 500 nM in all the solutions except for the measurements in ACN, where it was 5 μM. *λ*_ex_=480 nm. (**c**) Linear discrimination analysis (LDA) of 45 representative patterns generated by different analytes under diverse conditions. Initial conditions: m-SMS in EtOH-AcOH (10 mM) and ^i^3, ^ii^6, ^iii^8, ^iv^9 and ^v^11 mM of NaOH. DMSO, dimethylsulphoxide; dsDNA, double-stranded DNA; ssDNA, single-stranded DNA; THF, tetrahydrofuran.

The structure of m-SMS (Fig. 1a) consists of a *cis*-amino proline scaffold that is appended with three spectrally overlapping fluorophores: fluorescein (Flu), sulforhodamine B and nile blue (NB), which serve as a fluorescence resonance energy transfer (FRET) donor1–acceptor1/donor2–acceptor2 system, respectively. In addition, the sensor consists of various recognition elements for binding distinct chemical species. The boronic acid and dipicolylamine (DPA) groups, for example, provide m-SMS with an affinity towards different saccharides^45^ and metal ions^46^, respectively. The thiourea and sulfonamide functionalities serve as additional metal ion-binding sites^47^,^48^,^49^, as well as anion^50^ receptors and hydrogen-bonding motifs^51^,^52^. Additional binding interactions may involve hydrogen bonding with the amides and carboxylic acid of m-SMS, in addition to hydrophobic interactions and π-stacking with the various aromatic groups. Finally, the Flu structure and protonation state are highly pH dependent^53^, whereas solvatochromic NB^54^ can interact with DNA and hydrophobic analytes (Fig. 1a). Additional recognition sites could also be formed upon the binding of analytes. DPA–metal ion complexes, for example, are known to interact with anions such as phosphates^55^, whereas deprotonation of Flu by a base should enable the phenolic ligand to coordinate with metal ions^56^. This versatility of artificial receptors is counter intuitive to traditional fluorescent molecular sensor design^57^, because it aims at creating a sensor that is inherently nonspecific. In this way, the binding of different analytes should induce the formation of distinct emission signatures by affecting FRET, photo-induced electron transfer, dye conjugation or charge transfer processes^57^. For example, the binding of metal ions to DPA could disrupt or enhance photo-induced electron transfer^58^, whereas changes in pH or solvents could alter Flu conjugation^53^ or intramolecular charge transfer processes within NB. In addition, because the different signalling and recognition elements are integrated on a single molecular platform, the interaction of m-SMS with any chemical species is likely to change the distance between the probes, which would affect the FRET efficiency. This covalent integration of dyes should also facilitate hiding, sending and extracting the molecular device without affecting the molar ratio between them and consequently, without changing the device's photophysical properties.

### Multi-analyte identification

The unusual sensing mechanism underlying m-SMS was demonstrated by measuring its response to diverse chemical species (Fig. 1b) including different solvents (top left), metal ions (top right), saccharides (middle left), as well as its response to changing the pH (middle right) or polarity (bottom left) of the solution, and to the presence of complex mixtures such as those that can be found in soft drinks and medications (bottom right). Different emission signatures were also generated in the presence of different sugar phosphates, proteins and by changing analyte concentrations (Supplementary Figs 1 and 2). By analysing these patterns using linear discriminant analysis (LDA), which is an efficient pattern recognition algorithm for classifying unknown samples^59^, we could straightforwardly identify 45 representative analytes (Fig. 1c). Thirty-eight unknown samples that were randomly selected from the training set were identified by m-SMS with 97% accuracy.

### Molecular cryptography

**Figure 2:**
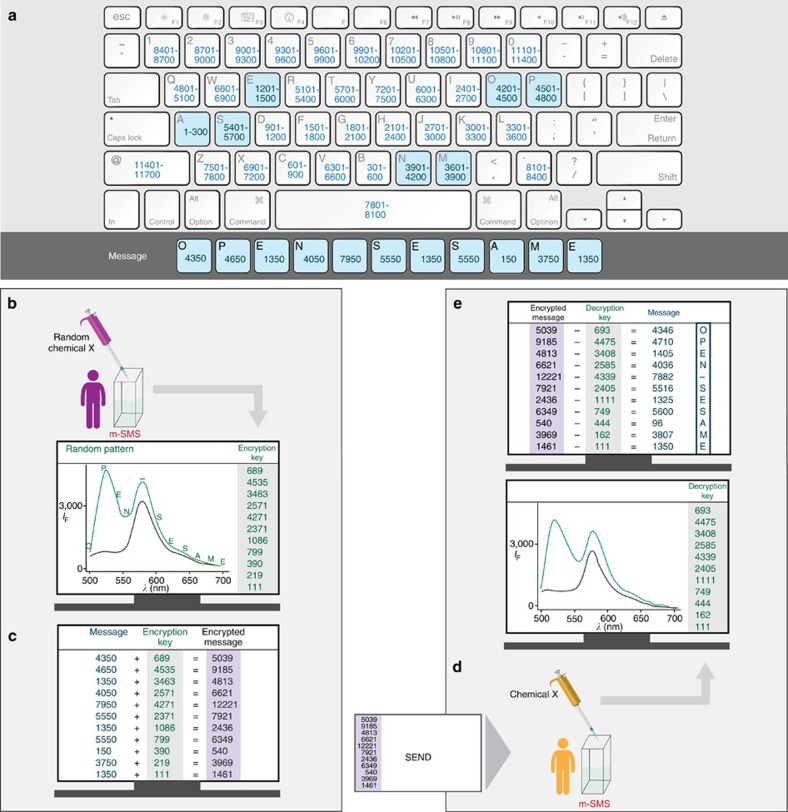
Cryptographic protection by an Enigma-like molecular cipher device. (**a**) The sender converts his message to numbers by using a public alphanumeric code. (**b**) He then dissolves m-SMS in a chosen solution, verifies the initial emission intensity (black line) and records the emission pattern generated after adding a random chemical input (green line). The resulting intensity values, recorded every 20 nm (denoted in green letters), provide a unique encryption key. (**c**) The sender then encrypts the message by adding the encryption key to the original message and sends the encrypted message (cipher text) to the recipient. (**d**) The recipient, who possesses an identical m-SMS cipher device, repeats this procedure by setting up the correct initial state of the system (for example, solvent, sensor concentration and detector gain) and adding the same chemical x. (**e**) The original message is then revealed by subtracting the resulting values (green line) from the cipher text. Conditions: 500 nM m-SMS in EtOH, chemical x=NaHCO_3_ (2 μl, 1 M), *λ*_ex_=480 nm. The following illustrations were used under a license from Shutterstock.com: keyboard (credit: Alhovik), pipette (credit: extender_01) and man character (credit: Leremy).

This ability of m-SMS to produce a wide range of nearly unpredictable emission fingerprints resembles the function of pseudo-random number generators, namely, cipher devices that can effectively encrypt text by associating each letter with an approximate random number. One of the most well-known pseudo-random number generator devices is the Enigma machine^60^,^61^, which was used by the Germans during World War II (WWII) to protect military communication. With the Enigma technology, the sender and receiver possessed identical cipher machines that were used to encrypt and decrypt the text, respectively. In addition, to prevent a third party with an identical machine from spying on these messages, the receiver must also have setup the correct initial state of his machine in order to obtain the right message. To elucidate the function of an Enigma-like molecular machine, we first show how m-SMS can be used to encrypt and decrypt a very simple text: ‘open sesame' (Fig. 2). Initially, the sender converts the text to numbers using a public alphanumeric code to obtain a numeric sequence (Fig. 2a). Note that this alphanumeric code does not need to be secure and can be used to write various other messages. In the next step, the sender dissolves m-SMS in a chosen solution (60 μl EtOH) to which 2 μl of a randomly selected chemical input (chemical *x*, 1 M NaHCO_3_) is added. A random encryption key is then generated by recording the emission every 20 nm and associating each value with the corresponding letter (Fig. 2b). The sender then adds this encryption key to the original message to afford an encrypted message (cipher text; Fig. 2c) that can be safely sent to a recipient with an identical molecular device. To obtain the original message, the receiver simply needs to generate the decryption key by setting up the correct initial state of the system (for example, sensor concentrations, solvents and detector gain), adding the same chemical input (Fig. 2d), and subtracting the resulting values from the cipher text (Fig. 2e).

**Figure 3:**
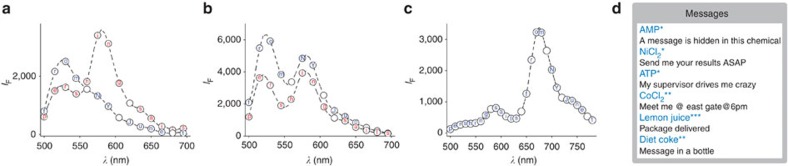
Encrypting longer messages by sequentially adding chemical inputs. (**a**) Encrypting a message by recording the emission spectra generated after adding NaOH (2 μl, 0.2 M, red letters) and then after adding CuCl_2_ (2 μl, 0.3 mM, blue letters) to 500 nM SMS in EtOH-AcOH (10 mM). (**b**) Encrypting the same message by recording the emission spectra after adding NaOH (2 μl, 0.35 M, red letters ) and then GenTeal eyedrop (2 μl, blue letters) to 500 nM SMS in EtOH-AcOH (10 mM). (**c**) Encrypting the same message by using a single, broad emission spectrum obtained after adding NaOH (0.5 μl, 0.35 M) and CuCl_2_ (1 μl, 0.3 mM) to 5 μM SMS in acetonitrile. These experiments (**a**–**c**) also demonstrate how the same message can be differently encrypted by changing the chemical inputs (**a** versus **b**) or by changing the initial state of the system (**a** versus **c**). (**d**) Representative messages that were successfully decrypted by untrained, randomly selected users. Initial conditions: m-SMS (500 nM) in *EtOH, **EtOH-AcOH (10 mM) and NaOH (6 mM), and ***EtOH-AcOH (10 mM) and NaOH (10 mM).

Figure 3 shows how longer messages can be encrypted by sequentially adding chemical inputs. For clarity, messages encrypted by two inputs are presented. The text ‘Pershing sails from NY June 1' was selected for this experiment because, in the context of hidden messages, this is a well-known message that was written by a spy during WWII.^2^ Hence, with this message, we intend to highlight the analogy between m-SMS and the simplest stereographic technologies in terms of the ease by which messages can be concealed and exposed by untrained users. In Fig. 3a, the encryption key was generated by first adding NaOH (0.2 M), then CuCl_2_ (0.3 mM) and recording the emission following each addition. In Fig. 3b, the inputs were changed to NaOH (0.35 M) and eyedrop, which demonstrate the feasibility of encrypting messages with commercially available chemicals. Pharmaceutical liquids are very suitable for this application owing to their high purity and batch-to-batch reproducibility, which enable the sender and receiver to use them as is without performing additional procedures. Figure 3c shows how an entirely different encryption key can be generated with the same inputs used in the first experiment (Fig. 3a, NaOH and CuCl_2_), but changing the solvent to acetonitrile and the concentrations of the molecular components to 5 μM m-SMS, 0.35 M NaOH and 0.3 M CuCl_2_. Owing to the stronger intensity of the NB dye under hydrophobic conditions, the message could be encrypted in a single emission spectrum, which was obtained after the second addition step. This last experiment (Fig. 3c) thus demonstrates the importance of correctly setting up the initial state of the system, which is a fundamental principle underlying the operation of Enigma machines^61^. Following these test cases, 12 different users, including 10 untrained users, were requested to decrypt different messages (2–19 words) by using different chemical inputs (Fig. 3d and Supplementary Table 1). The fact that all messages were successfully decrypted confirmed the simplicity, versatility and reliability of this technique.

### Molecular password protection

**Figure 4:**
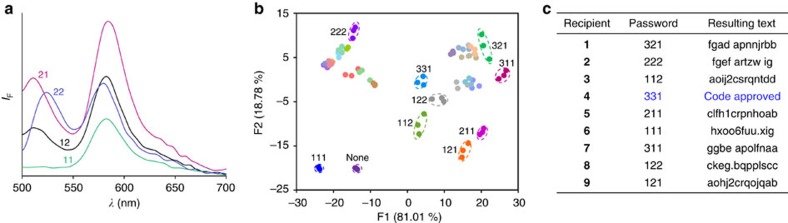
P**assword protection by generating sequence-dependent encryption keys.** By appropriately choosing chemical inputs, m-SMS can operate as a molecular keypad lock that generates the correct encryption/decryption keys (emission patterns) only when the chemical inputs are introduced in the right order. (**a**) Different encryption keys generated by introducing the four possible combinations of two-digit chemical ‘passwords' consisting of ZnCl_2_ (**1**) and Na_3_PO_4_ (**2**) as inputs signals. (**b**) LDA mapping of the encryption keys generated in response to the 27 possible combinations of three-digit chemical passwords, where ZnCl_2_ (**1**), Na_3_PO_4_ (**2**) and NaOH (**3**) serve as input signals. The clusters corresponding to the nine unique encryption keys are denoted in circles. Conditions: each digit corresponds to the addition of 2 μl of **1** (0.08 M), **2** (0.08 M) or **3** (0.1 M) to 60 μl m-SMS (500 nM) in EtOH. (**c**) Text obtained by decrypting the cipher text with the correct password (331) and by the other eight unique combinations.

Despite the fact that cryptography makes m-SMS far more secure than secret inks, there is always the possibility that the enemy would obtain the sensor and the correct chemical inputs, and would attempt to recreate the encryption key using a ‘brute force search'^2^. Namely, it would measure the response of m-SMS to different concentrations and combinations of these inputs until meaningful text would result from this screening. Figure 4 shows a means for complicating such efforts by entering a password as an additional layer of defence. This approach exploits the principles of molecular keypad lock technology^30^,^31^,^32^,^33^,^34^,^35^,^36^,^37^,^38^,^39^,^40^,^41^, which largely rely on the tendency of multivalent host–guest complexes and multicomponent assemblies to be entrapped in local minima^41^. We selected ZnCl_2_ (**1**), Na_3_PO_4_ (**2**) and NaOH (**3**) as representative entry keys owing to the strong interaction of Zn(II) with DPA ligands^55^,^56^, as well as with NaOH or Na_3_PO_4_ to yield Zn(OH)_2_ and zinc phosphate complexes, respectively^62^. Hence, when ZnCl_2_ is initially added, Zn(II) should readily coordinate to the DPA unit of m-SMS. In contrast, when ZnCl_2_ is added second, the reaction with an excess of Na_3_PO_4_ or NaOH in solution should reduce the concentration of free Zn(II) ions and consequently, the amount of the m-SMS-Zn(II) complex. Figure 4a exemplifies how m-SMS can be used to generate four different encryption keys using two-digit chemical passwords: 11, 22, 12 and 21. With three chemical inputs, additional metastable complexes can be formed, which enabled us to identify 9 unique passwords from the 27 possible combinations (Fig. 4b). The relevance of the keypad lock technique to cryptographic applications was demonstrated by providing nine different recipients with the same chemical inputs (1, 2 and 3), but with distinct individual passwords. As shown in Fig. 4c, only the receiver with the right password could successfully identify the message, whereas the other users only obtained random text.

### Molecular steganography

**Figure 5:**
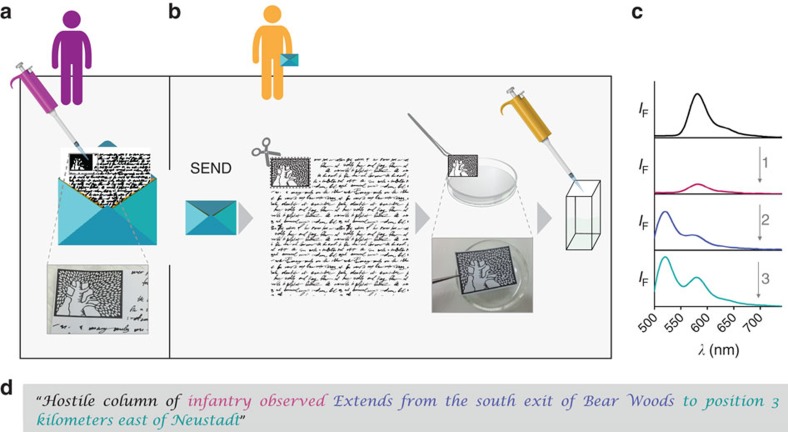
Steganographic protection by hiding m-SMS on plain letter paper. (**a**) 1.1 μl of m-SMS (440 μM) was hidden on a random spot within the logo of the Weizmann Institute and the letter was sent to a recipient by regular mail. Note that the text within this letter does not contain any valuable information. (**b**) The recipient, who obtained the cipher text and knows the initial conditions, extracts m-SMS from the paper by incubating the logo in 1 ml of EtOH-AcOH (10 mM). (**c**) To uncover the message, the receiver adjusts the correct concentration of m-SMS by calibrating its initial emission intensity (top) and generates the decryption key by recording the emission pattern following the addition of each chemical input (inputs 1–3). (**d**) The resulting text is a message that was encrypted by the Enigma machine. The letter colours correspond to relevant decryption keys shown in **c**. Conditions: 1 μl of (**1**) NiCl_2_ (0.15 M), (**2**) KOH (2.5 M) and (**3**) Na_4_EDTA (0.27 M) were sequentially added to a 60-μl solution of m-SMS (500 nM) in EtOH-AcOH (10 mM). The hand-writing text image (credit: amiloslava) is taken with permission from Shutterstock.com.

Steganography is the third layer of protection that can be implemented by concealing low quantities of m-SMS on regular paper (Fig. 5). This not only complicates its detection, but also its characterization, which would be needed if an enemy attempts to reproduce the molecular device. Figure 5 depicts a representative experiment in which 1.1 μl of m-SMS was dried on plain letter paper (Fig. 5a) and sent to a second recipient by regular postal services. In this experiment, the letter was printed with a standard printer and the sensor was hidden on a random spot within the logo of the Weizmann Institute (Fig. 5a). To clarify, the text within this letter does not contain any valuable information, but rather, the message is concealed within the emission spectra of m-SMS, which can only be generated by setting up the appropriate conditions. To reveal the message, the receiver merely needs to extract m-SMS from the letter by cutting the logo, incubating it in an appropriate solution, and use this solution to record the fluorescence spectra (Fig. 5b). By setting up the correct initial emission intensity (Fig. 5c, top spectrum) and sequentially adding the right chemical inputs (Fig. 5c, inputs 1–3), the receiver could successfully identify various different messages, such as the one presented in Fig. 5d: ‘Hostile column of infantry observed. Extends from the south exit of Bear Woods to position 3 kilometers east of Neustadt', a message that was encrypted by the original Enigma machine.

### Versatility of the m-SMS technology

**Figure 6:**
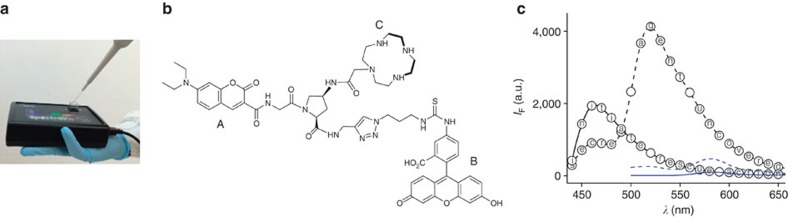
Versatility of the m-SMS technology. Secret communication was achieved by using (**a**) a hand-held spectrometer, and (**b**) a second molecular cipher device (m-SMS_2_) integrating coumarin (A), fluorescein (B) and a cyclen ligand (C). (**c**) Encryption patterns generated by m-SMS (blue lines) or m-SMS_2_ (black lines) under the same conditions. The emission of each sensor (250 nM) was recorded in EtOH solution containing NaOAc (1 mM) and ZnCl_2_ (1.3 mM; dashed line) and after adding AcOH (16 mM; solid line). m-SMS and m-SMS_2_ were excited at 480 and 420 nm, respectively.

Similar procedures, in which chemical inputs were concealed on letter paper, were also performed (Supplementary Fig. 3), demonstrating an alternative means of hiding and delivering molecular components. In these experiments, chemical inputs with measurable absorption spectra such as CoCl_2_ (Supplementary Fig. 3b,c) were extracted from the paper and, after determining their concentrations, were added to m-SMS. In addition to commercial chemicals, we also encrypted messages using unique inputs made in our laboratory^63^, which shows how messages can be further protected by using synthetic compounds that are difficult to characterize and reproduce (Supplementary Fig. 3a and Supplementary Tables 1 and 2). Finally, to demonstrate that this technology is not limited to particular locations or a specific sensor, we encoded and decoded messages outside the laboratory using a low-cost hand-held spectrofluorometer (Fig. 6a and Supplementary Fig. 4) and we also synthesized a second m-SMS molecule (Fig. 6b, m-SMS_2_) integrating coumarin and a pH-sensitive Flu probe, as well as a cyclen ligand that can bind various metal ions. Hence, similar to m-SMS (Fig. 1a), m-SMS_2_ (Fig. 6b) should be able to respond to metal ions, acids and bases. However, it should produce different emission patterns owing to the shorter excitation and emission wavelength of the FRET donor (that is, coumarin), as well as the distinct affinity of cyclen and DPA towards different metal ions. To demonstrate that this new molecular cipher device can generate entirely different encryption keys, the message ‘secret agent uncovered initiate rescue action' was encrypted by recording the emission of m-SMS_2_ before and after adding 16 mM acetic acid. We then attempted to decrypt the resulting cipher text by using both m-SMS_2_ and the original m-SMS. As shown in Fig. 6c, although the same chemical inputs were used, only the first molecular device successfully decrypted the messages. The second device generated a meaningless text. This last experiment thus shows that even if a third party manages to reproduce m-SMS and spy on the experimental settings, a new cipher device can be readily created by replacing one or several receptors, linkers or dyes.

## Discussion

Given recent concerns regarding global electronic surveillance^64^, the ability of m-SMS to convert different chemical structures into unique emission patterns demonstrates a potential means to bypass using electronic communication systems and thereby ensure that important messages are secure. Interestingly, even this first prototype provides a very high security level owing to its ability to generate numerous unpredictable encryption keys (cryptography), as well as the difficulty of finding and characterizing the molecular device and/or chemical inputs (steganography), and in particular cases, the order by which the inputs are introduced (password protection). In addition, as with Enigma cryptographic systems, to break such a defence one also needs to set up the correct initial state of the system, which can be determined by the type of solvents and concentrations used, as well as by the instrumentation setup. We can estimate, for example, the maximal number of patterns that can be generated by using six different concentrations of m-SMS (Supplementary Fig. 2a) at six different pH values (Supplementary Fig. 2b) and upon the addition of six different concentrations of copper ions (Supplementary Fig. 2c). By setting the detector to six different ‘gain' values (Supplementary Fig. 2d), even a single chemical input (that is, CuCl_2_), out of the numerous chemicals that can be discriminated by m-SMS (Fig. 1b), should afford a maximal number of 6^4^=1,296 encryption keys. Improving the performance of such systems should be readily achieved by increasing the number of recognition and signalling elements, which would maximize the number of analytes that can be discriminated by a unimolecular cipher device. Other important features of this technology, namely, its versatility and simplicity, have also been demonstrated by creating different m-SMS devices, encrypting messages with a wide range of randomly selected chemicals, as well as by hiding the molecular components on plain paper and sending them by regular mail, akin to invisible inks. Considering the unlimited number of chemical structures that can, in principle, be used as inputs, this work indicates that a unique message could be hidden within each and every molecule around us.

## Methods

### Synthesis and characterization of m-SMS and m-SMS_2_

Detailed synthesis and characterization of the m-SMSs are available in the Supplementary Methods.

### Multi-analyte sensing

Different analytes and their combinations were identified by adding them to m-SMS (500 nM) in an ethanol solution containing 10 mM of AcOH (EtOH-AcOH). In a typical experiment, a chemical input (2 μl) was added to 60 μl of m-SMS in EtOH-AcOH and the emission pattern was recorded by a BioTek synergy H4 hybrid multi-mode microplate reader (BioTek, Inc.) using black flat-bottom polystyrene 384-well microplates (Corning). This process was performed in four replicates and emission intensity values obtained at 520, 580 and 654 nm were analysed by LDA using XLSTAT version 2014.1.01. LDA reduces the dimensionality of the data into two canonical factors (F1 and F2), which enables classifying unknown samples according to the proximity of the data points (F1, F2) to the clusters obtained by the training set.

### Encryption and decryption of messages

Messages were ciphered and deciphered by adding one or several chemical inputs to m-SMS or m-SMS_2_ and recording the emission spectra with a BioTek synergy H4 hybrid multi-mode microplate reader or by using a portable SpectroVis Plus spectrophotometer (Vernier) connected to a laptop computer equipped with LoggerPro software. The intensity and shape of the spectral patterns, which provide the encryption/decryption keys, were varied by changing the chemical inputs and their concentrations, as well as by altering the initial state of the system. For example, different fluorescence fingerprints were readily obtained by changing the solvent, pH, photomultiplier gain (current amplification), sensor concentration and by combing of these parameters. In a typical experiment, generally, the encryption and decryption keys were generated by dissolving the molecular sensor (500 nM) in 60 μl EtOH or EtOH-AcOH (10 mM), adding 1–2 μl of chemical inputs, and recording the emission intensity values every 4–15 nm. This experiment was performed in triplicate. Steganographic protection was achieved by pipetting 1–2 μl of m-SMS or chemical inputs such as CoCl_2_ on the Weizmann Institute logo. The logo was printed on plain A4 paper by a standard HP colour LaserJet printer (M651). CoCl_2_ was extracted from the paper with 300 μl of water and its concentration was determined according to its extinction coefficient (*ɛ*_510 nm_=4.85 M^−1^ cm^−1^).

## Additional information

**How to cite this article:** Sarkar, T. *et al*. Message in a molecule. *Nat. Commun.* 7:11374 doi: 10.1038/ncomms11374 (2016).

## Supplementary Material

Supplementary Information Supplementary Figures 1-4, Supplementary Tables 1-3, Supplementary Methods and Supplementary References
