# Supplementary material for: Message in a molecule
Source: Nat Commun. 2016 May 3;7:11374. doi: 10.1038/ncomms11374 (PMC4857388; doi:10.1038/ncomms11374)
Supplement: Supplementary Information — Supplementary Figures 1-4, Supplementary Tables 1-3, Supplementary Methods and Supplementary References [file ncomms11374-s1.pdf]

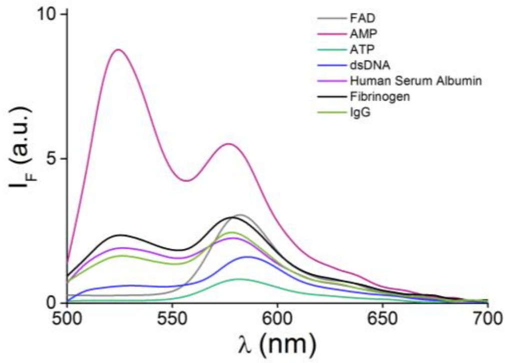

Supplementary Figure 1 │ Representative emission patterns generated by m-SMS in response to different sugar phosphates and proteins.

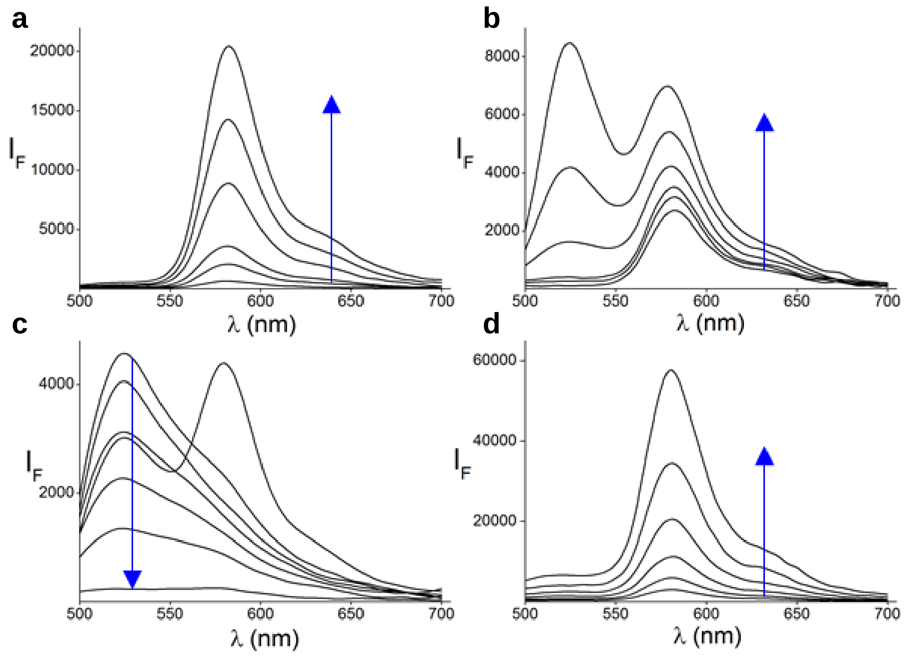

Supplementary Figure 2 │ (**a**) Distinct fluorescence patterns were generated using different concentrations (0.1, 0.3, 0.5, 1, 2, and 4 μM) of m-SMS in ethanol-AcOH (10 mM). Patterns generated upon the addition of 1 μL of (**b**) NaOH (3, 30, 60, 300, 420, and 480 mM) or (**c**) an aqueous solution of $CuCl_2$ (0, 0.04, 0.6, 40, 80, 120, and 200 mM) to a 60 μL solution of m-SMS in ethanol-AcOH (10 mM) or ethanol-AcOH (10 mM) and NaOH (6 mM), respectively. (**d**) Emission spectra obtained by setting the detector gain value to 100, 110, 120, 130, 140, and 150.

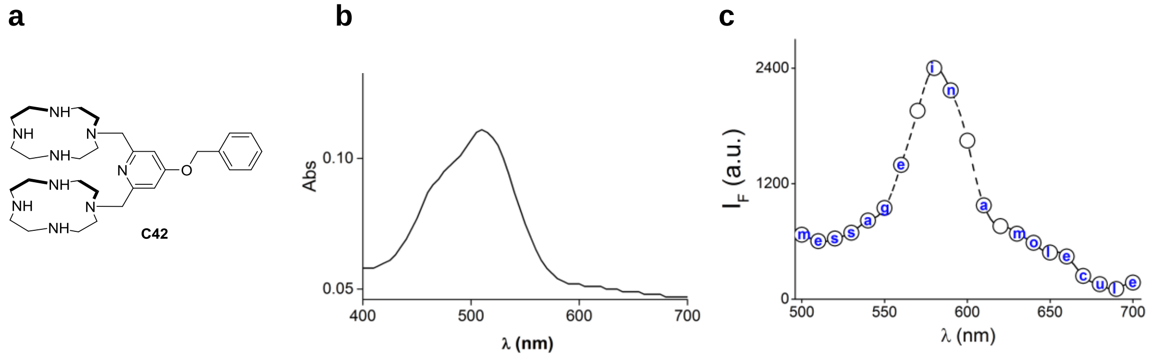

Supplementary Figure 3 │ (**a**) The structure of compound **C42**. (**b**) Absorption spectrum of $CoCl_2$, which was expected from letter paper. $CoCl_2$ was spotted and dried (4μL, 2 M) on letter paper and sent to a recipient. The recipient extracted the chemical ($CoCl_2$) from the paper by dissolving it in water (240-300 μL) and calibrated its concentration according to the absorption at 510 nm and an extinction coefficient of 4.85 M^{-1} cm^{-1}. (**c**) Decryption key generated by adding the extracted chemical input (1 μL $CoCl_2$, 35 mM) to 60 μL m-SMS (500 nM) in EtOH-AcOH (10 mM) and NaOH (6 mM).

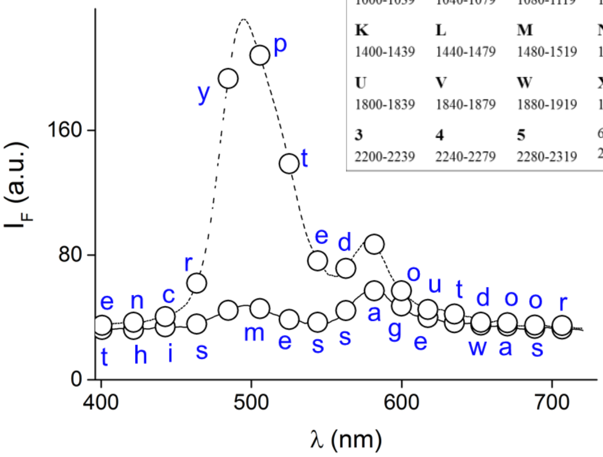

**A** 1000-1039	**B** 1040-1079	**C** 1080-1119	**D** 1120-1159	**E** 1160-1199	**F** 1200-1239	**G** 1240-1279	**H** 1280-1319	**I** 1320-1359	**J** 1360-1399
**K** 1400-1439	**L** 1440-1479	**M** 1480-1519	**N** 1520-1559	**O** 1560-1599	**P** 1600-1639	**Q** 1640-1679	**R** 1680-1719	**S** 1720-1759	**T** 1760-1799
**U** 1800-1839	**V** 1840-1879	**W** 1880-1919	**X** 1920-1959	**Y** 1960-1999	**Z** 2000-2039	**space** 2040-2079	**.** 2080-2119	**1** 2120-2159	**2** 2160-2199
**3** 2200-2239	**4** 2240-2279	**5** 2280-2319	6 2320-2359	**7** 2360-2399	**8** 2400-2439	**9** 2440-2479	**0** 2480-2519	**@** 2520-2559	

Supplementary Figure 4│Encrypting a message using a hand-held spectrofluorometer. The encryption key was generated by recording the emission spectrum of m-SMS (2.2 μM) in ethanol (600 μL) before (solid line) and after adding 1 μL $NaHCO_3$ (0.98 M) (dashed line). Inset: the corresponding alphanumeric code.

Supplementary Table 1. A list of messages encrypted using m-SMS.

Entry	Input	Message	Spectral Range (nm)	Interval (nm)
1[a]	ATP	my supervisor drives me crazy	546-658	4
2[b]	$NiCl_2$	send me your results ASAP	500-740	10
3[a]	AMP	a message is hidden in this chemical	500-710	6
4[c]	ascorbic acid	my sensor is working	500-614	6
5[c]	lemon Juice	package arrived	500-640	10
6[b]	NaOH	pershing sails	500-695	15
	$CuCl_2$	from NY June 1	500-695	15
7[b]	NaOH	pershing sails	500-695	15
	eyedrop	from NY June 1	500-695	15
8[e]	$CuCl_2$	pershing sails from NY June 1	500-780	10
9[a]	ATP	message in a bottle	542-650	6
10[d]	m-SMS in EtOH-AcOH	hostile column of	560-624	4
	$NiCl_2$	infantry observed	560-624	4
	KOH	extends from south exit of Bear Woods	500-660	4
	Na_4EDTA	to position 3 kilometers east of neustadt	500-656	4
11[b]	$CoCl_2$	meet me @ east gate @6pm	500-644	6
12[a]	$NiCl_2$	Searching SciFinder	500-680	10
13[c]	Nescafe	come @science museum @8pm	500-740	10
14[d]	NaOH	leaving to position 5	500-660	8
15[d]	diet Coke	message in a bottle	500-644	8
16[b]	$CoCl_2$	message in a molecule	500-700	10
17[f]	C42	the code has been broken	550-642	4
18[g]	none	this message was	400.6-700.7	each 20 points
	$NaHCO_3$	encrypted outdoor	400.6-720.6	

m-SMS in [a]EtOH, [b]EtOH-AcOH (10 mM) and NaOH (6 mM), [c]EtOH-AcOH (10 mM) and NaOH (10 mM), and [d]EtOH-AcOH (10 mM), [e] m-SMS (5 µM) in ACN and NaOH(11 mM), [f]m-SMS in EtOH and Na_3PO_4 (65 µM); the concentration of C42 was 40 µM, [g]m-SMS (2.2 µM) in EtOH, this message was generated using a hand-held spectrophotometer (I_F= 2000 AU).

Supplementary Table 2. List of encryption keys, cipher texts and decryption keys corresponding to the messages presented in Supplementary Table 1.

Message 1			Message 2			Message 3		
encryption key	cipher text	decryption key	encryption key	cipher text	decryption key	encryption key	cipher text	decryption key
374	4124	423	525	6075	497	990	1140	968
456	7806	498	758	2108	747	2012	9962	1938
561	8511	607	941	4991	982	3642	7392	3634
744	6294	780	910	1960	954	5039	6389	4854
1035	7185	1040	845	4595	861	5647	11197	5564
1415	6065	1381	810	2160	794	5367	10917	5217
1786	3136	1782	925	8275	867	4626	4776	4477
2110	7360	2160	1385	5735	1262	3715	5665	3676
2366	8816	2454	1525	7675	1556	3140	4490	3066
2447	4997	2576	1405	6655	1314	2889	10839	2767
2368	7918	2479	1005	6255	939	3133	5683	2959
2168	6518	2232	760	2110	629	3759	9309	3737
1912	7162	1924	546	6096	478	4463	12413	4325
1604	9554	1632	482	6632	399	4698	6948	4665
1340	2390	1380	337	3787	380	4437	6987	4501
1143	6393	1194	258	6108	199	3684	4734	3763
934	3484	1025	198	5748	232	3025	4075	2811
783	7233	881	174	324	57	2274	3624	2095
662	2012	781	105	5655	124	1744	5794	1706
620	6170	715	115	265	89	1326	9276	1282
612	8562	663	62	4712	55	1253	3803	1195
579	4329	636				1132	5182	1019
568	1918	610				996	8946	988
501	8451	543				870	6720	911
431	1181	494				752	3002	650
397	5647	439				607	3157	608
385	535	417				405	5955	462
310	7960	380				487	8437	321
239	7589	368				297	1047	157
						246	2496	310
						231	1581	236
						158	3908	226
						100	2650	32
						147	897	34
						254	404	79
						99	3549	101

Message 4			Message 5			Message 6		
encryption key	cipher text	decryption key	encryption key	cipher text	decryption key	encryption key	cipher text	decryption key
266	4016	283	448	5098	383	600	5250	677
299	7649	320	488	638	482	1495	2845	1516
340	8290	360	538	1288	557	1617	6867	1634
366	5916	383	581	3731	560	1390	6940	1373
364	1714	389	574	724	557	1814	4064	1761
349	4399	379	606	2556	520	3403	5953	3480
342	5892	371	595	1945	589	3121	7171	3159
337	4687	385	749	8699	853	1785	3735	1785
355	5605	395	864	1014	951	1075	9025	1128
384	8334	423	736	5986	855	863	6413	901
465	3015	473	547	5797	621	547	697	595
560	6110	583	394	2944	435	272	2822	289
684	8634	729	323	6773	351	150	3600	187
767	7517	849	325	1675	268	203	5753	180
771	5121	876	241	1291	283			
706	5956	791				796	2446	936
595	3745	647				2159	7409	2305
476	3026	529				2504	6854	2529
372	4422	423				1928	5678	1949
299	2249	349				1552	9502	1562
						1323	5373	1299
						964	8314	981
						573	8523	622
						418	3268	415
						259	6409	404
						189	4239	116
						73	1423	120
						42	7992	89
						38	8588	6

**Message 7**			**Message 8**			**Message 9**		
encryption key	cipher text	decryption key	encryption key	cipher text	decryption key	encryption key	cipher text	decryption key
1196	5846	1225	122	4772	125	364	4114	382
3619	4969	3731	207	1557	203	413	1763	435
3169	8419	3293	265	5515	258	561	6111	624
1900	7450	1972	293	5843	287	882	6432	969
2242	4492	2122	295	2545	296	1415	1565	1494
3933	6483	3852	321	2871	321	1959	3909	2063
3367	7417	3375	374	4424	375	2366	3716	2433
1792	3742	1815	568	2518	561	2430	10380	2486
1126	9076	1109	766	8716	765	2168	4718	2219
942	6492	933	800	6350	815	1751	5801	1774
552	702	589	659	809	639	1340	9290	1381
354	2904	352	528	3078	533	1038	1188	1025
227	3677	245	458	3908	461	783	8733	872
156	5706	119	463	6013	440	637	1087	740
			687	8637	669	612	4962	708
2096	3746	1996	1258	2908	1243	580	6430	606
5487	10737	5573	2344	7594	2336	501	6351	521
5924	10274	6002	3226	7576	3220	388	3838	432
3825	7575	3739	3215	6965	3252	385	1735	374
3376	11326	3297	2637	10587	2593			
4849	8899	4943	2028	6078	2008			
4426	11776	4391	1453	8803	1474			
2547	10497	2415	1133	9083	1132			
1589	4439	1516	1066	3916	1050			
1215	7365	1093	1030	7180	1089			
835	4885	693	890	4940	862			
534	1884	638	734	2084	748			
323	8273	311	529	8479	513			
175	8725	240	410	8960	477			

Message 10								
encryption key	cipher text	decryption key	encryption key	cipher text	decryption key	encryption key	cipher text	decryption key
1042	3292	1047	841	2191	826	1127	6977	1153
1502	5852	1495	1439	8489	1404	1969	6319	1986
2002	7552	1983	1913	7763	1862	2638	10588	2646
2455	8305	2423	2263	3613	2198	3134	7784	3133
2799	5349	2754	2489	6539	2413	3455	7805	3447
2968	6418	2913	2591	3641	2508	3603	9153	3589
2947	4297	2875	2525	8075	2430	3508	6058	3483
2753	10703	2695	2320	10270	2226	3230	9080	3201
2452	3202	2395	2046	3696	1956	2844	5394	2817
2091	6441	2057	1752	7002	1669	2450	6800	2417
1741	5191	1719	1490	5840	1428	2092	6142	2071
1453	7603	1424	1281	5031	1232	1803	9753	1779
1222	4972	1188	1131	9081	1095	1593	10743	1579
1041	5091	1011	1044	6894	1019	1465	4615	1459
914	8864	901	994	3244	974	1429	3979	1417
824	5174	813	972	2322	950	1475	4925	1451
767	2417	759	987	8937	952	1590	5940	1542
			1012	6562	962	1739	5489	1665
			1020	5370	955	1895	3245	1780
315	1362	328	1013	7163	925	2000	7850	1877
401	1896	419	971	6821	889	2027	3377	1904
480	2463	514	913	3163	821	1977	7227	1851
557	2980	602	833	8783	743	1851	7401	1737
617	3370	672	746	2096	667	1672	9622	1573
657	3571	713	643	7693	584	1457	2807	1377
655	3531	716	567	3117	509	1248	1398	1178
623	3318	684	491	6341	458	1064	6614	1007
577	2972	621	425	8375	413	914	6764	864
503	2559	540	382	4732	373	785	8735	751
437	2156	462	346	1996	333	692	5042	659
368	1792	383	316	8266	301	632	2282	590
308	1496	325	300	750	279	596	8546	554
262	1274	274	281	1631	258	571	4621	518
224	1126	244	260	410	237	533	1883	493
198	1011	219	237	5487	219	499	6649	459
191	949	206	219	8169	192	451	6001	431
			188	6938	176	402	6252	395
			175	4525	156	353	503	362
			167	4517	142	301	1351	313
			163	1213	128	269	6119	279
			143	5693	128			

Message 11			Message 12			Message 13		
encryption key	cipher text	decryption key	encryption key	cipher text	decryption key	encryption key	cipher text	decryption key
300	4050	337	525	6075	497	1680	2430	1664
358	1708	354	758	2108	747	3758	8108	3934
406	1756	405	941	1091	982	6102	9852	6101
437	6287	456	910	6160	954	6123	7473	6061
448	8398	464	845	1595	861	5052	13002	4946
438	4188	449	810	3060	794	4053	15603	3988
416	1766	425	925	3475	867	3812	9362	3718
403	8353	416	1385	5435	1262	4758	5508	4655
412	11962	425	1525	3475	1556	5245	7795	5178
455	8405	457	1405	9355	1314	4502	5852	4431
548	1898	543	1005	6555	939	3298	7348	3244
710	860	710	760	1510	629	2301	3051	2198
885	6435	918	546	3096	478	1780	3130	1740
999	6849	1072	482	2132	399	1568	9518	1550
1006	8956	1076	337	2887	380	1305	5055	1274
903	2853	952	258	4308	199	989	7139	1005
747	897	765	198	1248	232	785	6335	776
572	6422	607	174	1524	57	615	3165	636
449	1799	468	105	5355	124	389	6539	351
367	8317	382				458	4208	545
316	11866	331				378	8328	337
269	8219	291				215	11765	173
236	10286	250				198	10848	278
220	4870	235				125	4775	168
201	3951	210				201	3951	154

Message 14			Message 15			Message 16		
encryption key	cipher text	decryption key	encryption key	cipher text	decryption key	encryption key	cipher text	decryption key
531	3981	540	204	3954	253	669	4419	894
693	2043	677	250	1600	300	602	1952	660
831	981	838	285	5835	280	629	6179	648
847	7297	955	311	5861	325	690	6240	612
818	3368	864	294	444	358	816	966	750
746	4796	847	299	2249	335	947	2897	846
765	2715	799	353	1703	341	1395	2745	1512
778	8728	871	395	8345	438	1956	9906	1776
1132	6982	1096	574	3124	621	2400	4950	2730
1487	5837	1531	749	4799	855	2168	6218	2136
1770	9720	1833	910	8860	982	1647	9597	1776
1642	6292	1756	830	980	911	973	1123	1008
1287	5637	1345	668	8618	728	758	8708	546
935	6485	947	555	1005	517	679	4429	468
703	3253	656	374	4724	382	586	4936	546
584	6434	497	324	6174	357	483	3933	276
495	3045	494	326	6176	303	443	1793	816
448	4798	412	233	3683	272	241	991	126
360	4410	323	152	1502	206	154	6304	522
256	8206	223				104	3554	60
248	9998	208				172	1522	444

Message 17			Message 18		
encryption key	cipher text	decryption key	encryption key	cipher text	decryption key
134	5984	168	31	1811	32
181	2431	215	30	1330	33
238	1588	284	35	1375	32
288	8238	368	33	1773	32
465	1215	538	42	2102	36
603	4953	654	48	1548	39
766	1816	818	38	1218	36
845	2195	944	37	1777	33
894	8844	986	42	1782	35
844	3094	958	56	1076	43
813	963	859	48	1308	38
634	6184	736	40	1220	36
611	8561	682	35	2095	33
453	903	528	34	1934	33
433	1783	449	33	1053	32
300	1650	436	33	1773	32
336	4386	381			
256	8206	254	35	1175	36
265	715	285	37	1217	36
238	5488	271	40	1140	39
228	4578	223	54	1754	52
235	3385	254	160	2140	142
215	1565	173	223	1843	218
155	4205	199	157	1937	168
			85	1265	91
			68	1208	69
			89	2149	87
			57	1637	57
			45	1865	46
			40	1820	40
			36	1176	36
			35	1615	35
			34	1614	35
			33	1733	32

Supplementary Methods

General Information

All chemicals, sugars, metal salts, and anhydrous solvents were obtained from Sigma Aldrich. Glycoseptol mouth wash, GenTeal eye drops, and Otrivin nasal spray were purchased from a local pharmaceutical store, and diet Coke, lemon juice (Sicilia), Nescafe, wine, Carlsberg beer, and BLU energy drink were obtained from a local grocery store. ssDNA and dsDNA stands for $^{5'}$GTCACGTCATAGCTGCCTCTACTGTCGATCGTCGTA$^{3'}$ and A20.T20, respectively, which were obtained from W. M. Keck Foundation Biotechnology at Yale University. Deuterated solvents were purchased from Cambridge Isotope Laboratories, Inc. or Armar Chemicals. Anhydrous solvents were transferred using an oven-dried syringe. Flasks were oven-dried under a stream of argon. The ^{1}H NMR and ^{13}C NMR spectra were recorded on a Bruker Avance 300 MHz, a Bruker Avance 400 MHz, or an Agilent VNMRS 600 MHz spectrometer. The chemical shifts are represented in ppm on the δ scale down field from TMS as the internal standard. The following abbreviations were used to describe the peaks: s-singlet, d-doublet, t-triplet, q-quartet, and m-multiplet. Electrospray ionization mass spectral data (ESI-MS) were recorded on a Micromass Platform LCZ-4000 instrument at the Weizmann Institute of Science mass spectrometry facility. Flash column chromatography was performed on an automated Combiflash Teledyne ISCO instrument. The analytical reversed-phase high-performance liquid chromatography (RP-HPLC) data were obtained on an Agilent Technologies 1260 Infinity HPLC (equipped with diode array UV detector and autosampler) using a C_{18} column. m-SMS was purified by preparative RP-HPLC using a Thermo Scientific separation instrument (P200 pump, UV 100 detector), and a pre-packed Vydac C_{18} column. Fluorescence measurements were carried out using a BioTek synergy H4 hybrid multiwell plate reader and using black flat-bottom polystyrene NBS 384-well microplates (Corning). For the experiments using a handheld spectrophotometer the spectra were acquired using a SpectroVis Plus spectrophotometer (Vernier). Linear discriminant analysis was performed using XLSTAT version 2014.1.01. The following illustrations in the Figures 1 and 5 were used under license from Shutterstock.com: keyboard (credit:

Alhovik), pipette (credit: extender_01), hand-writing text (credit: amiloslava) and man character (credit: Leremy).

Abbreviations

ACN	acetonitrile
AcOH	acetic acid
AMP	adenosine monophosphate
ATP	adenosine triphosphate
DCM	dichloromethane
DIEA	N,N'-diisopropylethylamine
DMF	N,N'-dimethylformamide
DMSO	dimethyl Sulfoxide
DPA	di-(2-picolyl)amine
dsDNA	double strand DNA
EDC	1-ethyl-3-(3-dimethylaminopropyl)carbodiimide
EtOH	ethanol
FAD	flavin adenine dinucleotide
HATU	1-[bis(dimethylamino)methylene]-1H-1,2,3-triazolo[4,5-b]pyridinium 3-oxid hexafluorophosphate
HOBT	hydroxybenzotriazole
LDA	linear discriminant analysis
Na_4EDTA	tetrasodium ethylenediaminetetraacetic
ssDNA	single strand DNA
TFA	trifluoroacetic acid
THF	tetrahydrofuran

Synthesis of m-SMS

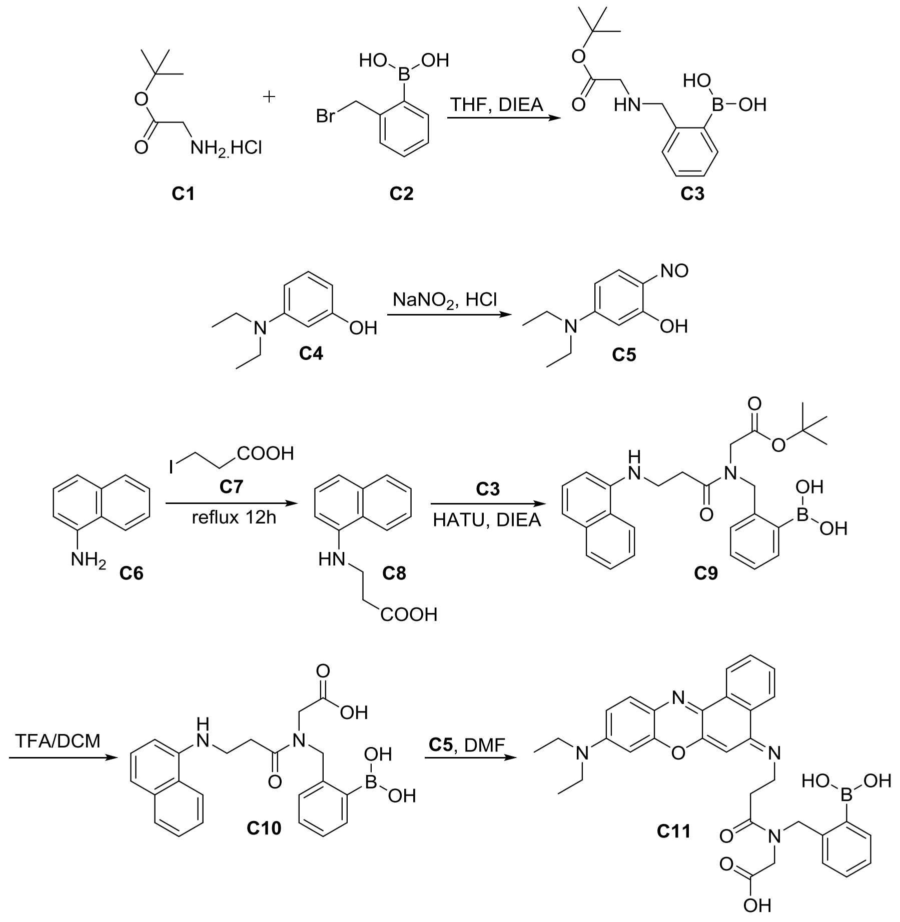

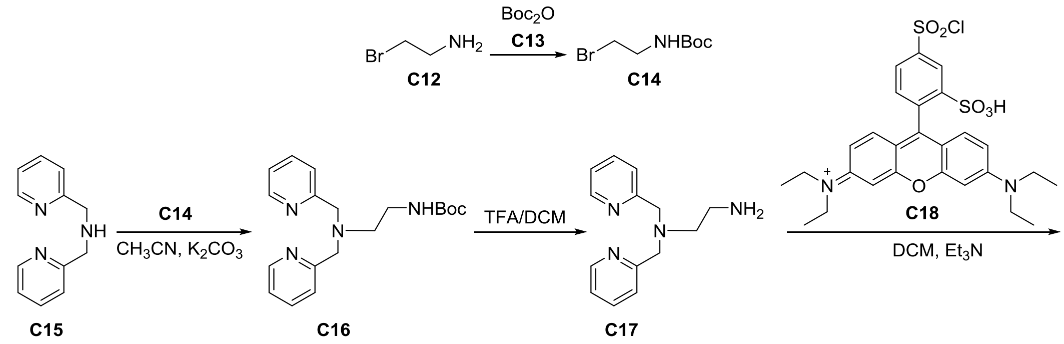
Boc2O
C13
Br NH2
Br NHBoc
C12
C14
SO2Cl
SO3H
C14
CH3CN, K2CO3
NH
NHBoc
TFA/DCM
NH2
C18
DCM, Et3N
C15
C16
C17

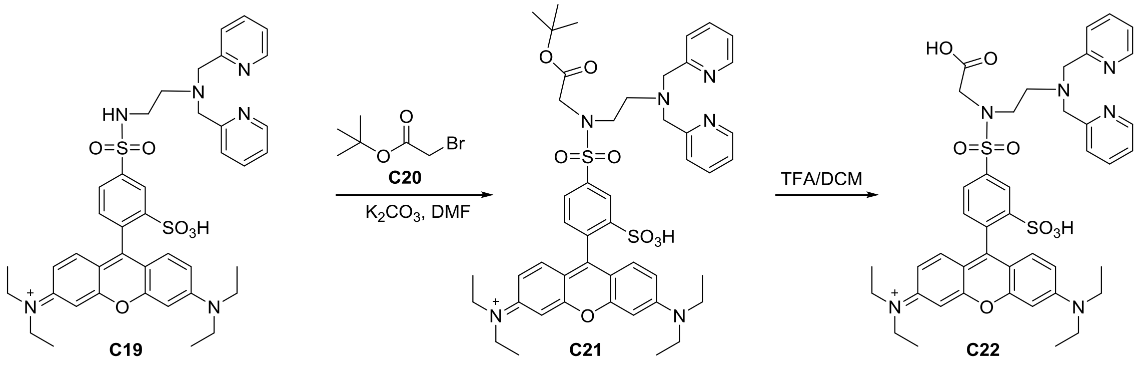
HN
O=S=O
SO3H
C19
C20
K2CO3, DMF
Br
C21
TFA/DCM
HO
C22

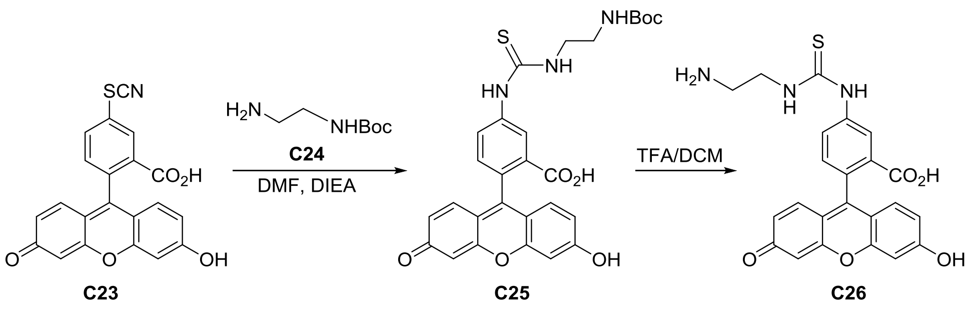
SCN
CO2H
OH
C23
H2N
NHBoc
C24
DMF, DIEA
NHBoc
C25
TFA/DCM
H2N
C26

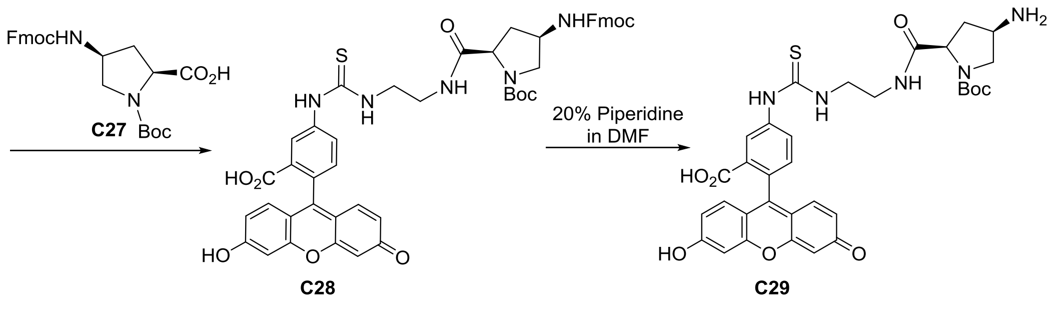
FmocHN
CO2H
C27
Boc
NHFmoc
HO2C
C28
20% Piperidine
in DMF
NH2
C29

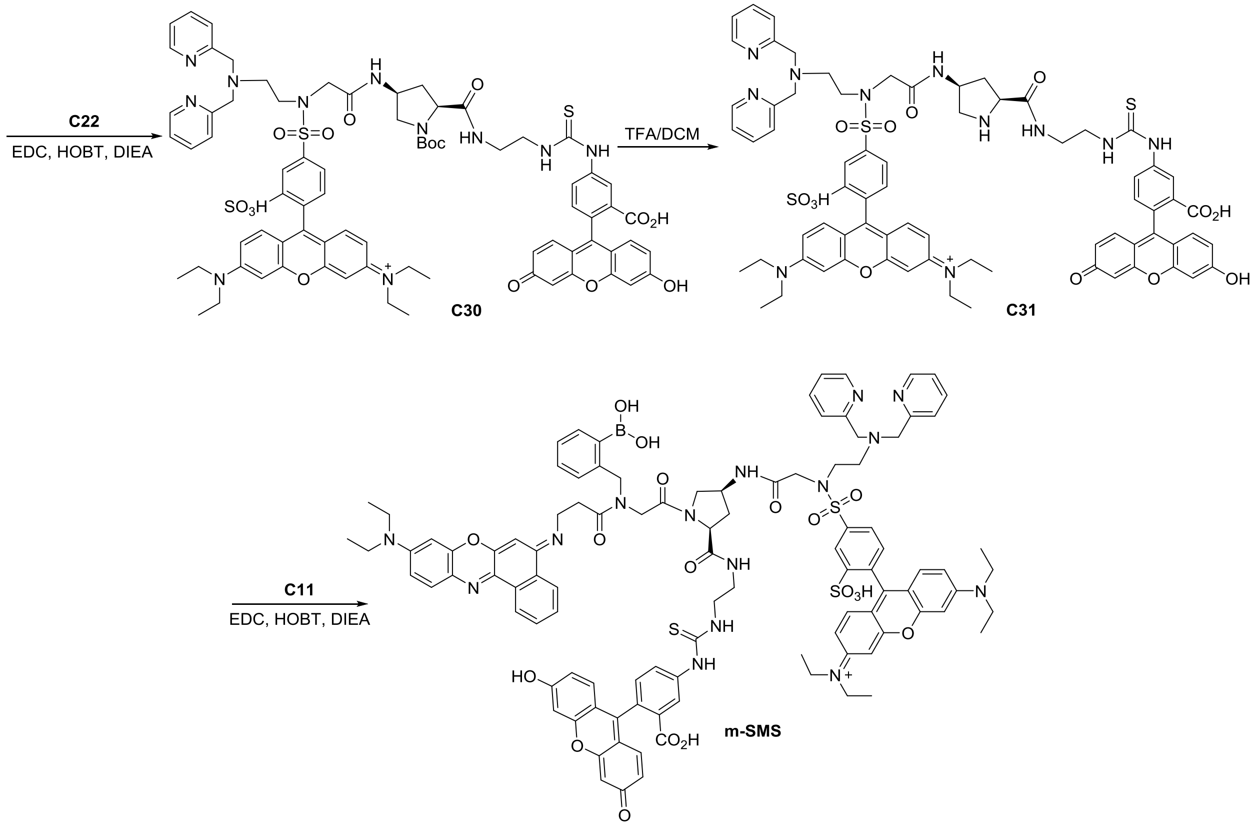
C22
EDC, HOBT, DIEA
TFA/DCM
C30
C31
C11
EDC, HOBT, DIEA
m-SMS

Synthesis of m-SMS_2

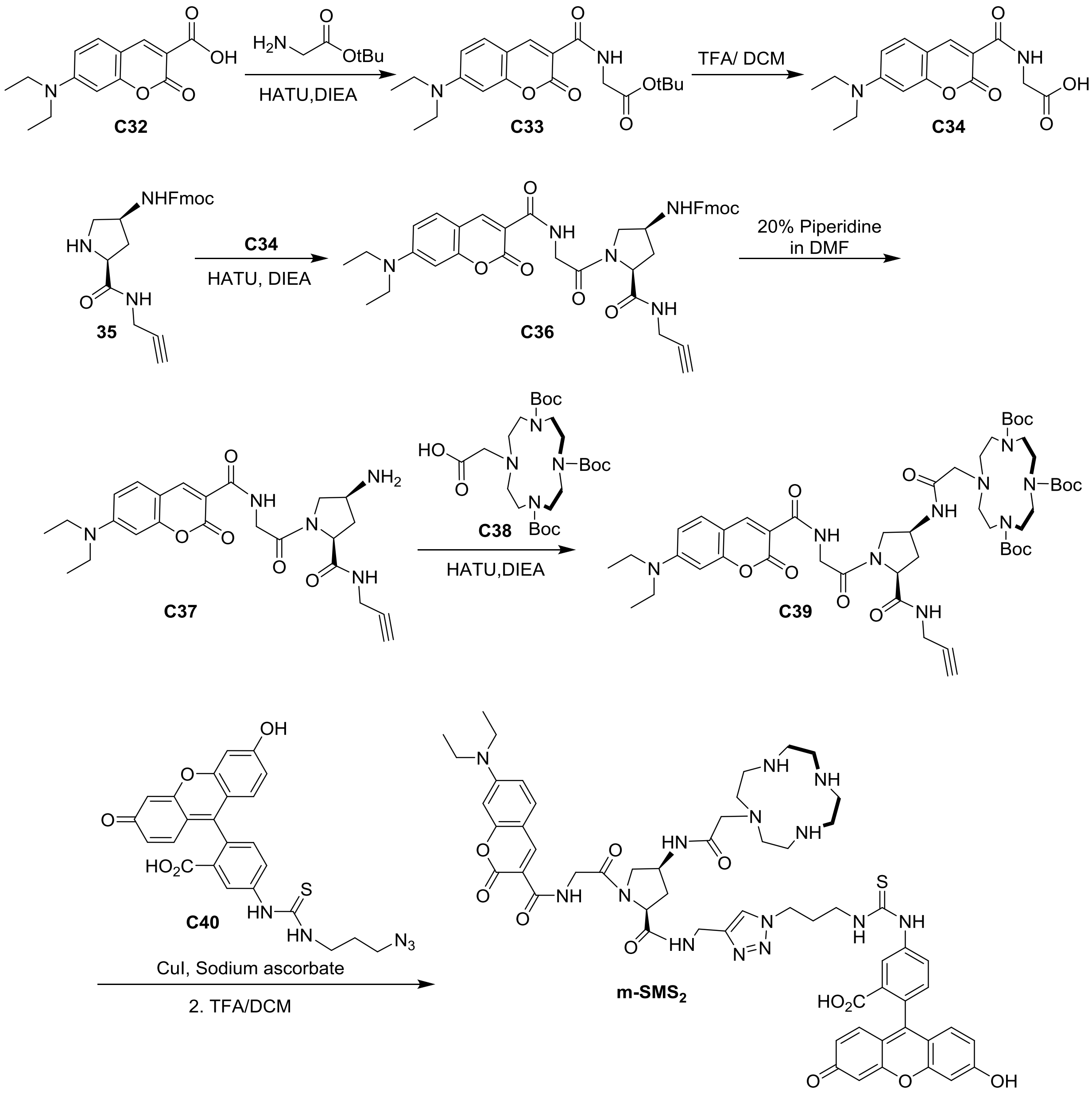

Synthetic Procedures

Compound C3: To a stirred solution of **C1** (tert-butyl glycinate) (0.4 g, 3.05 mmol) in dry THF (20 mL) were added **C2** (2-bromomethyl phenyl boronic acid) (0.59 g, 2.77 mmol) and triethylamine (0.8 mL, 6.08 mmol) under argon. The solution was refluxed at 70 °C for 8 h. Thereafter, the solvent was evaporated, the residue was dissolved in DCM/ MeOH (4:1 v/v, 50 mL) and washed twice with water (50 mL) and then with brine (20 mL). The organic layer was dried over anhydrous sodium sulfate. Then the solvent was evaporated to dryness and the crude reaction mixture was subjected to combi-flash column chromatography (silica gel, 7.2% MeOH in DCM) to obtain **C3** (0.56 g) as a white solid in 76% yield. ^{1}H NMR (300 MHz, CD_3OD): δ = 7.45-7.43 (t, *J* = 6 Hz, 1H), 7.23- 7.21 (t , *J* = 6 Hz, 2H), 7.17- 7.15 (br m,1H), 4.15 (s, 2H), 3.56 (s, 2H), 1.55 (s, 9H); ^{13}C NMR (75 MHz, CD_3OD): δ = 168.33, 141.19, 130.04, 127.15, 126.33, 122.63, 82.40, 54.44, 49.02, 35.0, 29.28. ^{13}C NMR (75 MHz, CD_3OD): 168.3, 170.7, 130.0, 127.1, 126.3, 82.5, 54.3, 29.4, 26.8. HRMS-ESI (*m/z*): calcd. for $[M+Na+2MeOH-2H_2O]^+$ 316.1696; found 316.1690.

Compound C8: Compound **C7** (3-iodopropanoic acid) (0.502 g, 2.5 mmol) was added to a stirred solution of **C6 (**naphthalen-1-amine) (0.3 g, 2.1 mmol) in dry EtOH (20 mL). Then the reaction mixture was stirred for 12 h under argon at 80 °C. After the reaction was completed, solvent was evaporated and the residue was dissolved in ethyl acetate (50 mL), washed twice with water (50 mL) and then with brine (20 mL). The organic layer was dried over anhydrous sodium sulfate and evaporated to dryness. The crude product was subjected to combi-flash column chromatography (silica gel, 25% EtOAc in hexane) to yield **C8** (0.26 g) in 58% yield.^{1}H NMR (300 MHz, CD_3OD): δ = 7.97-7.95 (m, 1H), 7.83-7.71 (m, 1H), 7.40- 7.38 (t, *J* = 3 Hz, 2H), 7.32-7.27 (t, *J* = 6 Hz, 1H), 7.16 (d, *J* = 9 Hz, 1H), 6.63 (d, *J* = 6 Hz, 1H), 3.77-3.72 (t, *J* = 6 Hz, 2H), 2.76- 2.72 (t, *J* = 6 Hz, 2H); ^{13}C NMR (75 MHz, $CDCl_3$) δ = 177.5, 142.1, 138.5, 134.3, 128.6, 126.4, 125.9, 125.1, 119.9, 118.6, 105.3, 39.7, 33.2. HRMS-ESI (*m/z*): calcd. for $[M+H]^+$ 216.1025; found 216.1023.

Compound C9: A solution of compound **C8** (0.178 g, 0.83 mmol) in DCM (4 mL) was cooled to 0 °C. Then, HATU (0.34 g, 0.91 mmol) and a solution of compound **C3** in

DCM/DMF (3:1 v/v, 2 mL) were added. After adding DIEA (0.11 mL, 0.83 mmol), the reaction mixture was stirred at room temperature for 18h under argon. After the reaction was completed and monitored by TLC, the solvent was removed in vacuum and the crude material was subjected to column chromatography (silica gel, 30% EtOAc in hexane) to afford **C9** (0.169 g) in a 49% yield. ^{1}H NMR (300 MHz, $CDCl_3$): δ = 7.90 (d, *J* = 6 Hz, 1H), 7.78 (d, *J* = 9 Hz, 1H), 7.47-7.42 (m, 3H), 7.37- 7.32 (t, *J* = 9 Hz, 2H), 7.27-7.24 (m, 3H), 6.65 (d, *J* = 6 Hz, 1H), 6.25 (br s, 1H), 3.94 (s, 2H), 3.92 (s, 2H), 3.66-3.62 (t, *J* = 6 Hz, 2H), 2.69- 2.66 (t, *J* = 6 Hz, 2H), 1.46 (s, 9H); ^{13}C NMR (75 MHz, CD_3OD) δ = 173.9, 143.1, 139.3, 134.5, 131.7, 131.5, 128.8, 128.3, 127.9, 126.9, 126.2, 125.2, 124.0, 123.8, 120.4, 116.71, 103.7, 81.8, 52.5, 39.7, 31.7, 31.5, 26.8. HRMS-ESI (*m/z*): calcd. for $[M+Na+2MeOH-2H_2O]^+$ 513.2537; found 513.2531.

Compound C11: Trifluoroacetic acid (1 mL) was added to a solution of compound **C9** (0.18 g, 0.38 mmol) in DCM (1 mL) at 0 °C. Then the reaction mixture was allowed to warm up to room temperature and stirring was continued for 2 h. After the reaction was completed, DCM and TFA were evaporated and the residue was dried under high vacuum for 6 h. This crude mass of compound **C10** is sufficiently pure for use in the next step. The crude solid **C10** (0.13 g; 0.32 mmol) was dissolved in DMF (10 mL). After adding compound **C5**[1] (0.63 g, 0.326 mmol), the reaction mixture was heated to 70 °C and stirred for 5 h. After removing the solvent, the crude material was purified by combi-flash column chromatography (silica gel, 5% MeOH in DCM) to afford crystalline blue solid of **C11** (0.110 g, 58%). ^{1}H NMR (300 MHz, CD_3OD): δ = 8.70-8.63 (m, 1H), 8.32 (d, *J* = 6 Hz, 1H), 8.22 (d, *J* = 6 Hz, 1H), 7.82-7.78 (m, 1H), 7.74-7.67 (t, *J* = 6 Hz, 2H), 7.33-7.29 (br, 2H), 7.23-7.18 (, t, *J* = 6 Hz, 2H), 6.83 (s, 1H), 6.76 (br s, 1H), 4.73 (s, 2H), 4.01 (s, 2H), 3.97- 3.95 (br m, 2H), 3.70- 3.67 (t, *J* = 3 Hz 4H), 3.06-2.95 (br m , 2H), 1.37-1.32 (t, *J*=6 Hz, 6H). ^{13}C NMR (75 MHz, CD_3OD) δ = 173.9, 171.8, 156.9, 154.1, 150.5, 147.53, 134.7, 132.5, 131.2, 130.2, 129.8, 128.8, 127.8, 126.3, 123.6, 122.7, 122.6, 115.5, 115.4, 95.2, 93.2, 52.6, 47.3, 40.5, 33.9, 30.7, 11.1. HRMS-ESI (*m/z*): calcd. for for $[M+Na+2MeOH-2H_2O]^+$ 631.2704; found 631.2703.

Compound C14: To a solution of **C12 (**2- bromoethylamine hydrobromide) (1 g, 4.9 mmol) in dry THF (25 mL) was added dropwise a solution of **C13 (**di-*t*-butyl

dicarbonate) (1.1 g, 4.9 mmol) in THF (15 mL) followed by Et_3N (0.77 mL, 5.39 mmol). After stirring the mixture for 8h, the solvent was evaporated. Then the residue was dissolved in DCM/MeOH (4:1 v/v, 50 mL) and washed twice with water (50 mL) and then with brine (20 mL). The organic layer was dried over anhydrous sodium sulfate. Then the solvent was evaporated to dryness and the crude product was subjected to combi-flash column chromatography (silica gel, 5% hexane in EtOAc) to obtain the desired product **C14** (0.56 g) as a white solid in 52% yield. ^{1}H NMR (300 MHz, $CDCl_3$): δ = 5.06 (br s, 1H), 3.62 (t, *J* = 3 Hz, 2H), 3.42 (q, *J* = 3 Hz, 2H), 1.50 (s, 9H). ^{13}C NMR (75 MHz, $CDCl_3$) δ = 161.1, 65.0, 41.5, 40.4, 27.1. MS-ESI (*m/z*): calcd. for $[M+Na]^+$ 246.01; found 246.06.

Compound C16[2]: Compound **C15** (0.5 g, 2.51 mmol) in anhydrous CH_3CN (20 mL) was refluxed in the presence of K_2CO_3 (0.59 g, 2.51 mmol) and potassium iodide (0.095 g, 0.57 mmol) under argon for 30 min. Then **C14** (0.618 g, 2.76 mmol) was added and the reaction mixture was refluxed for 6h. After the reaction was completed, the solvent was evaporated and the residue was dissolved in ethyl acetate (50 mL) and washed twice with water (50 mL) and then with brine (20 mL). The organic layer was dried over anhydrous sodium sulfate and evaporated to dryness. The crude was subjected to combi-flash column chromatography (silica gel, 2% MeOH in DCM) to afford **C16** (0.48 g) in a 55% yield. ^{1}H NMR (300 MHz, $CDCl_3$): δ = 8.55 (d, *J* = 6 Hz, 2H), 7.66-7.61 (t, *J* = 6 Hz, 2H), 7.42 (d, *J* = 6 Hz, 2H), 7.17-7.13 (t, *J* = 6 Hz, 2H), 5.81 (br s, 1H), 3.78 (s, 4H), 3.24-3.22 (m, 2H), 2.74-2.70 (t, *J* = 6 Hz, 2H), 1.43 (s, 9H). ^{13}C NMR (75 MHz, $CDCl_3$): δ = 148.6, 136.9, 136.8, 123.6, 122.5, 122.4, 59.5, 55.8, 50.9, 30.9, 27.8. MS-ESI (*m/z*): calcd. for $[M+Na]^+$, 365.19; found 365.22, calcd. for $[2M+Na]^+$ 707.40; found 707.46.

Compound C19: A solution of compound C**16** (0.3 g, 0.87 mmol) in 50% TFA in DCM (2 mL) was stirred for 3h at room temperature. After the reaction was completed, DCM and TFA were evaporated and the residue was dried in vacuum to afford compound **C17**[2] as a sticky solid. Compound **C17** (0.187 g, 0.774 mmol) was dissolved in dry DCM (8 mL) and the solution was basified to pH 7-8 with Et_3N (0.18 mL, 1.6 mmol). This solution was added dropwise to a stirred solution of **C18** (Lissamine Rhodamine B Sulfonyl Chloride) (0.4 g, 0.69 mmol) in dry DCM (20 mL). After adding Et_3N (0.14

mL), the stirring was continued for 12h. After the reaction was completed and monitored by TLC, solvent was evaporated and the crude material was purified by combi-flash column chromatography (silica gel, 9% MeOH in DCM) to afford compound **C19** (0.21 g, 39%).^{1}H NMR (300 MHz, $CDCl_3$): δ = 8.86 (s, 1H), 8.67 (d, *J* = 6 Hz, 2H), 8.06 (dd, *J* = 6 Hz, *J* = 9 Hz, 1H), 7.90-7.85 (t, *J* = 6 Hz, 2H), 7.53 (d, *J* = 9 Hz, 2H), 7.35-7.31 (t, *J* =6 Hz, 2H), 7.22-7.18 (m, 3H), 6.80 (dd, *J* = 9 Hz, *J* = 12 Hz, 2H), 6.67 (d, *J* = 3 Hz, 2H), 4.13 (s, 4H), 3.58-3.52 (br m, 8H), 3.28-3.24 (t, *J* = 6 Hz, 2H), 3.07-3.03 (t, *J* = 6 Hz, 2H), 1.32-128 (t, *J* = 6 Hz, 12H). MS-ESI (*m/z*): calcd. for $[M+Na]^+$ 805.28; found 805.14, calcd. for $[M+2Na]^{2+}$ 414.13; found 414.17.

Compound C21: To compound **C19** (0.20 g, 0.255 mmol) in anhydrous DMF (5 mL) were added **C20** (tert-butyl 2 bromoacetate) (0.054 g, 0.28 mmol) and K_2CO_3 (0.128 mg, 0.541 mmol) under argon. The reaction mixture was stirred for 12h. The solvent was evaporated and the crude was purified by combi-flash column chromatography (silica gel, 6% MeOH in DCM) to afford **C21** as a pinkish solid (0.15 g) in 66% yield. ^{1}H NMR (300 MHz, $CDCl_3$): δ = 8.72 (br s, 2H,), 8.07- 8.03 (t, *J* = 3 Hz, 2H), 7.96- 7.89 (m, 2H), 7.49- 7.45 (t, *J* = 6 Hz, 2H), 7.30- 7.21 (m, 5H), 6.85 (d, *J* = 9 Hz, 2H), 6.62 (br s, 2H), 4.41 (s, 4H), 4.10 (s, 2H), 3.69- 3.64 (t, *J* = 6 Hz, 2H), 3.60- 3.50 (br q, 8H), 3.30-3.26 (t, *J* = 6 Hz, 2H), 1.42 (s, 9H), 1.33-128 (t, *J* = 6 Hz, 12H). HRMS-ESI (*m/z*): calcd. for $[M+Na]^+$ 919.3493; found 919.3463. The *tertiary* ester group of compound **C21** was deprotected as follows: trifluoroacetic acid (1 mL) was added to a solution of **C21** (0.15 g, 0.016 mmol) in DCM (1 mL) at 0 °C. Then the reaction mixture was allowed to warm up to room temperature and stirring was continued for 2h. After the reaction was completed, DCM and TFA were evaporated and the residue was dried under high vacuum for 6h to obtain compound **C22**.

Compound C25: To a stirred solution of **C23** (fluorescein isothiocyanate) (0.2 g, 0.512 mmol) in dry DMF (5 mL) were added **C24** (tert-butyl (2-aminoethyl)carbamate) (0.09 g, 0.563 mmol) and DIEA (0.106 mL, 6.14 mmol) under argon. The solution was stirred for 12h under dark conditions. After removing the solvent, the residue was re-dissolved in DCM/MeOH (4:1 v/v, 50 mL) and washed twice with water (50 mL) and then with brine (20 mL). The organic layer was dried over anhydrous sodium sulfate and evaporated to

dryness. The organic layer was evaporated and the crude material was purified by combi-flash column chromatography (silica gel, 2.8% MeOH in DCM) to obtain **C25** (0.12 g, 53%) as a white solid. ^{1}H NMR (300 MHz, CD_3OD): δ = 8.08 (s, 1H), 7.76 (dd, *J* = 3 Hz, *J* = 6 Hz 1H), 7.18 (s, 1H), 7.15 (br s, 1H), 6.76 (d, *J* = 9 Hz, 1H), 6.68 (d, *J* = 3 Hz, 1H), 6.58 (d, *J* = 3 Hz, 1H), 6.55 (d, *J* = 3 Hz, 2H), 3.74- 3.70 (t, *J* = 6 Hz, 2H), 3.33-3.31 (br t, 2H), 1.41 (s, 9H). ^{13}C NMR (75 MHz, CD_3OD): δ = 181.5, 175.0, 170.0, 160.9, 158.5, 157.4, 152.8, 140.7, 128.9, 124.4, 119.2, 118.8, 112.8, 110.4, 78.9, 60.5, 44.2, 39.0, 27.4. HRMS-ESI (*m/z*): calcd. for $[M + H]^+$ 550.1682; found 550.1648.

Compound C28: Trifluoroacetic acid (1 mL) was added to a solution of compound **C25** (0.122 g, 0.22 mmol) in DCM (1 mL) at 0 °C. Then the reaction mixture was allowed to warm up to room temperature and stirring was continued for 2h. After the reaction was completed, DCM and TFA were evaporated and the residue was dried under high vacuum for 6h. The crude solid was dissolved in DCM/DMF (9:1 v/v, 10 mL) and the solution was basified to pH 7-8 with DIEA (0.17 mL, 0.9 mmol). Then **C27** (N-Boc-cis-4-N-Fmoc-amino-L-proline) (0.091 g, 0.20 mmol), EDC (0.034 g, 0.22 mmol), and HOBT (0.037 g, 0.24 mmol) were added and the reaction mixture was stirred at room temperature for 18h under argon under dark conditions. The solvent was removed in vacuum and the crude was purified by combi-flash column chromatography (silica gel, 72% EtOAc in hexane) to afford **C28** as a yellow crystalline solid (0.088 g, 50%). ^{1}H NMR (300 MHz, CD_3OD): δ = 8.10 (s 1H), 7.79- 7.72 (m, 3H), 7.61 (d, *J* = 6 Hz, 2H), 7.39- 7.35 (t, *J* = 6 Hz, 2H), 7.29 (d, *J* = 6 Hz, 2H), 7.12 (d, *J* =6 Hz, 1H), 6.66 (d, *J* = 6 Hz, 4H), 6.53 (d, *J* = 6 Hz, 2H), 4.31 (s, 2H), 4.18- 4.16 (br m, 3H), 3.85- 3.74 (m, 3H), 3.60- 3.53 (t, 2H), 2.56 (br s, 1H), 2.00-1.92 (br t, *J* = 6 Hz, 2H), 1.44 (s, 9H). ^{13}C NMR (100 MHz, CD_3OD): 169.6, 156.6, 152.5, 151.8, 144.3, 144.2, 140.9, 140.7, 135.0, 134.3, 128.9, 128.6, 127.6, 127.4, 126.8, 124.8, 124.4, 123.9, 119.6, 112.3, 112.1, 109.5, 101.8, 80.6, 79.7, 66.3, 59.7, 59.6, 38.7, 27.51, 28.2, 27.2. HRMS-ESI (*m/z*): calcd. for $[M + Na]^+$ 906.2785; found 906.2802.

Compound C30: First, compound C**28** (0.08 g, 0.09 mmol) was dissolved in 20% piperidine in DMF (1.5 mL). The reaction mixture was stirred for 2h at room temperature under dark conditions. After the reaction was completed, DMF (4 mL) was added and the

mixture was extracted with hexane (3 × 10 mL) to remove piperidine. Then DMF was removed under high vacuum at 40 °C to obtain the yellowish compound **C29**. Compound **C22** was dissolved in DCM/DMF (9:1 v/v, 10 mL) and the solution was basified to pH 7-8 with DIEA (0.17 mL, 0.9 mmol). Then, compound **C29** (0.04 g, 0.06 mmol), EDC (0.011 g, 0.073 mmol), and HOBT (0.012 g, 0.078 mmol) were added and the reaction mixture was stirred at room temperature for 18h under argon and under dark conditions. The solvent was removed in vacuum and the crude was purified by combi-flash column chromatography (silica gel, 12% DCM in methanol) to afford **C30** (0.04 g, 44%). ^{1}H NMR (300 MHz, CD_3OD): δ = 8.51- 8.39 (br m, 2H), 8.26-8.17 (m, 1H), 8.00 (d, *J* = 3 Hz, 1H), 7.75- 7.72 (m, 4H), 7.66-7.64 (m, 4H), 7.34 (d, *J* = 6 Hz, 4H), 7.11- 7.05 (br m, 2H), 6.86 (d, *J* = 9 Hz, 1H), 6.73 (br s, 1H), 6.65-6.62 (m, 4 H), 6.51 (d, *J* = 6 Hz, 2H), 4.23- 4.05 (m, 3H), 3.92 (br s, 2H), 3.81 (s, 2H), 3.31 (d, *J* = 3 Hz, 4H), 3.19 (d, *J* = 3Hz, 3H), 2.92 (br s, 1H), 2.84 (br m, 2H), 2.15-2.13 (m, 8 H), 2.03- 1.94 (m, 2H), 1.60 (m, 1H,), 1.41-1.36 (t, *J* = 9 Hz, 12H,), 1.20 (s, 9H,). MS-ESI (*m*/z): calcd. for $[M-H]^-$ 1483.50; found 1483.67, calcd. for $[M-2H]^{2-}$ 741.51; found 741.12.

Compound m-SMS: Compound **C30** (0.03 g, 0.02 mmol) was dissolved in 50% TFA in DCM (1:1 v/v, 2 mL) and the mixture was stirred at room temperature for 4h. After the reaction was completed, DCM and TFA were evaporated and the residue was dried under high vacuum to afford compound **C31** as a sticky solid. **C31** was dissolved in dry DMF (3 mL) and the solution was basified to pH 7-8 with DIEA (0.18 mL, 1.6 mmol). This solution was added dropwise to a stirred solution of **C11** (0.11 g, 0.019 mmol) in dry DMF (1 mL). After adding EDC (0.003 g, 0.018 mmol) and HOBT (0.021 g, 0.003 mmol), the reaction mixture was stirred for 12h. After removing the solvent, the crude was purified on a preparative RP-HPLC using mobile phase A (0.1% TFA in H_2O) and B (0.1% TFA in ACN/H_2O, 3:1, v/v) to afford a bluish-violet solid (4 mg, 10%). The purity of m-SMS was analyzed by analytical RP-HPLC. ^{1}H NMR (600 MHz, CD_3OD): δ = 9.02 (d, *J* = 6 Hz, 1H), 8.74 (d, *J* = 6 Hz, 3H), 8.50 (s, 2H), 8.40 (s, 1H), 8.35 (d, *J* = 6 Hz, 1H), 8.2 (s, 1H), 8.10-8.03 (m, 3H), 8.0-7.97 (t, *J* = 6 Hz, 2H), 7.88- 7.87 (t, *J* = 6 Hz, 3H), 7.67 (d, *J* = 12 Hz, 2H), 7.59-7.55 (m, 3H), 7.43 (d, *J* = 6Hz, 1H), 7.27-7.26 (m, 1H), 7.14- 7.05 (m, 3H), 7.0 (br s, 1H), 6.94-6.90 (m, 4H), 6.92-6.59 (m, 4H), 6.51-6.50 (t, *J* = 6 Hz, 3H), 4.51 (br s, 6H), 4.09-3.95 (m, 5 H) 3.81-3.64 (br s, 14 H), 3.45 (br m,

1H), 3.2 (s, 2H), 3.14-3.13 (t, *J* = 6 Hz, 2H), 2.82 (s, 2H), 2.69-2.68 (m, 2H), 2.13-2.11 (m, 2H), 2.05 (s, 2H), 2.01-1.95 (t, *J* = 3 Hz, 2H), 1.28 (br m, 18 H,). HRMS-ESI (*m/z*): calcd. for [(M+2MeOH-2H_2O)] 1973.7212; found 1973.7263.

Compound C33. Compound **C32** (0.5 g, 1.9 mmol) and glycine tert-butyl ester hydrochloride (0.39 g, 2.3 mmol) were dissolved in THF (20 mL). To this mixture, DIEA (0.68 mL, 3.82 mmol) and HATU (0.87 g, 2.29 mmol) were subsequently added at room temperature. The brown reaction mixture turned into a clear yellow solution after 30 min. The reaction mixture was stirred for 3h at room temperature. Then, the solvent was evaporated. The obtained solid was purified by flash chromatography using hexane/ethyl acetate (1:1) to afford compound **C33** as a yellow solid (0.63 g, 88%). ^{1}H NMR (300 MHz, $CDCl_3$) δ = 9.18 (t, *J* = 4.9 Hz, 1H), 8.69 (s, 1H), 7.42 (d, *J* = 8.9 Hz, 1H), 7.26 (s, 1H), 6.65 (dd, *J* = 8.9, 2.3 Hz, 1H), 6.51 (d, *J* = 2.1 Hz, 1H), 4.12 (d, *J* = 5.4 Hz, 2H), 3.45 (q, *J* = 7.1 Hz, 4H), 1.49 (s, 9H), 1.23 (t, *J* = 7.1 Hz, 6H). ^{13}C NMR (75 MHz, $CDCl_3$) δ = 168.85, 163.35, 162.61, 157.82, 152.58, 148.32, 131.28, 110.15, 108.56, 96.94, 82.07, 45.30, 42.56, 28.17, 12.49. HRMS-ESI (*m/z*): calcd. for $[M+Na]^+$ 397.1739; found 397.1740.

Compound 36. Trifluoroacetic acid (2.5 mL) was added to a solution of compound **C33** (0.63 g, 1.68 mmol) in DCM (2.5 mL). The reaction mixture was stirred overnight at room temperature. The solvent and TFA were then evaporated to yield an oily liquid. Addition of ethanol to the oily liquid afforded a bright yellow solid. Then it was filtered and washed with ethanol (5 mL), followed by diethyl ether (5 mL). The solid was vacuum dried to afford **C34** (0.39 g, 73%). This compound was used in the next step without further characterization. Compound **C34** (0.39 g, 1.23 mmol) and compound **C35**[3] (0.48 g, 1.23 mmol) were dissolved in THF (15 mL). Then DIEA (0.42 mL) and HATU (0.56 g, 1.47 mmol) were added. The reaction mixture was stirred for 4h at room temperature. The solvent was evaporated. The residue was dissolved in DCM (20 mL), passed through a sintered glass filter containing a pad of neutral alumina and eluted with DCM (100 mL). The eluted fraction was evaporated and purified by a combi-flash column chromatography, using 1% methanol in DCM to afford compound **C36** as a bright yellow solid (0.41g, 48%). ^{1}H NMR (300 MHz, $CDCl_3$) δ = 9.41 (t, *J* = 4.5 Hz,

1H), 8.67 (s, 1H), 7.91 (d, *J* = 4.8 Hz, 1H), 7.75 (d, *J* = 7.4 Hz, 2H), 7.65-7.57 (m, 2H), 7.40 (dd, *J* = 12.6, 5.7 Hz, 2H), 7.35-7.27 (m, 2H), 7.05 (d, *J* = 6.9 Hz, 1H), 6.71 – 6.58 (m, 1H), 6.50 (s, 1H), 4.79 (d, *J* = 8.7 Hz, 1H), 4.53-4.38 (m, 1H), 4.35-4.07 (m, 6H), 3.99-3.87 (m, 1H), 3.74 (dt, *J* = 31.7, 7.8 Hz, 2H), 3.44 (q, *J* = 13.5, 4H), 2.56-2.22 (m, 2H), 2.20 (s, 1H), 1.24 (t, *J* = 6.9 Hz, 6H). HRMS-ESI (*m/z*): calcd. for $[M+Na]^+$ 712.2747; found 712.2762.

Compound C39. Compound **C36** (0.33 g, 0.49 mmol) was deprotected using 20% piperidine in DMF (1 mL). After 1 h, diethyl ether (30 mL) was added and the resulting precipitate was filtered and washed several times with diethyl ether and twice with hexane (10 mL). The obtained yellow solid (**C37**) was dried under vacuum and used in the next step. Compound **C37** (0.13 g, 0.28 mmol) and C**38**[4] (0.15 g, 0.28 mmol) were placed in a round bottomed flask. Then, THF (5 mL), DIEA (0.06 mL), and HATU (0.13 g, 0.34 mmol) were subsequently added. The resulting mixture was stirred overnight at room temperature. The solvent was evaporated and the residue was dissolved in DCM (50 mL). The reaction mixture containing DCM was washed twice with water and dried over sodium sulfate. The organic layer was evaporated and purified on an alumina column using and 1% methanol in DCM to afford a yellow solid (0.12 g, 44%). ^{1}H NMR (300 MHz, $CDCl_3$) δ = 9.37 (t, *J* = 4.6 Hz, 1H), 8.65 (s, 1H), 7.97 (s, 1H), 7.86 (s, 1H), 7.41 (d, *J* = 9.0 Hz, 1H), 6.63 (dd, *J* = 8.9, 1.9 Hz, 1H), 6.49 (d, *J* = 1.7 Hz, 1H), 4.75 (d, *J* = 8.1 Hz, 1H), 4.60 (dd, *J* = 8.1, 4.5 Hz, 1H), 4.35-3.69 (m, 6H), 3.65-3.46 (m, 6H), 3.44 (q, *J* = 14.2 Hz, 4H), 3.32 (s, 6H), 3.24 (d, *J* = 2.7 Hz, 2H), 2.84 (s, 4H), 2.35 (d, *J* = 13.7 Hz, 1H), 2.23 (s, 1H), 2.22 (s, 1H), 1.45 (s, 9H), 1.43 (s, 18H), 1.22 (t, *J* = 7.0 Hz, 6H). ^{13}C NMR (75 MHz, $CDCl_3$) δ = 171.21, 170.01, 169.45, 163.48, 162.50, 157.80, 156.02, 155.50, 152.71, 148.32, 131.24, 109.97, 109.59, 108.27, 96.62, 79.59, 79.24, 79.05, 71.63, 59.83, 54.91, 54.22, 53.45, 49.72, 49.05, 47.68, 45.09, 42.42, 31.49, 29.43, 28.68, 28.51, 12.42. HRMS-ESI (*m/z*): calcd. for $[M+Na]^+$ 1002.5276; found 1002.5262.

Compound m-SMS$_2$. Compound **C39** (30 mg, 31 µmol) and **C40**[5] (30 mg, 61 µmol) were dissolved in DMF/ DCM (4 mL; 1:1). Then, acetic acid (2.5 µL) and triethyl amine (5 µL) were added. After degassing the solution with argon, CuI (1 mg, 5µmol) was added under argon. Then, a solution of sodium ascorbate (2 mg, 10 µmol) in H_2O (100

μL) was added via a syringe needle. The reaction mixture was stirred under argon for 18h. After removing the solvent, the crude was purified by RP- HPLC. Then, the boc groups were removed using 30% TFA in DCM (2 mL). After evaporation of TFA and DCM, the crude reaction mixture was purified by RP-HPLC to afford m-SMS_2 (2 mg, 6%). ^{1}H NMR (500 MHz, MeOD) δ 8.63 (s, 1H), 8.23 (s, 1H), 8.13 (s, 1H), 7.68 (d, *J* = 8.6 Hz, 1H), 7.55 (d, *J* = 8.9 Hz, 1H), 7.14 (d, *J* = 7.9 Hz, 1H), 6.84 (d, *J* = 8.7 Hz, 1H), 6.72 (s(br), 3H), 6.62 (s, 1H), 6.58 (d, *J* = 8.1 Hz, 2H), 4.34-4.30 (m, 1H), 3.94 (dd, *J* = 10.1, 5.6 Hz, 1H), 3.68 (s, 4H), 3.55 (q, *J* = 6.8 Hz, 4H), 3.47 (s, 2H), 3.40 (s, 2H), 3.22 (s, 4H), 3.18 (s, 8H), 2.95 (br s, 6H), 2.58-2.52 (m, 1H), 2.38-2.30 (m, 2H), 2.18-2.15(m, 1H), 1.34-1.30 (m, 2H), 1.25 (t, *J* = 6.7 Hz, 6H). HRMS-ESI (*m/z*): calcd. for $[M+H]^+$ 1169.4991; found 1169.5023.

Supplementary References

1. Pheeney, C.G. & Barton, J.K. Intraduplex DNA-mediated electrochemistry of covalently tethered redox-active reporters. *J. Am. Chem. Soc.* **135**, 14944-14947 (2013).
2. Hirayama, T., Taki, M., Kodan, A., Kato, H. & Yamamoto, Y. Selective labeling of tag-fused protein by tryptophan-sensitized luminescence of a terbium complex. *Chem. Commun.* **22**, 3196-3198 (2009).
3. Selvakumar, K., Motiei, L. & Margulies, D. Enzyme−artificial enzyme interactions as a means for discriminating among structurally similar isozymes. *J. Am. Chem. Soc.* **137**, 4892-4895 (2015).
4. Jeon, J.W. et al. Protein-cleaving catalyst selective for protein substrate. *Org. Lett.* **4**, 4155-4158 (2002).
5. Barnard, A. et al. Orthogonal functionalisation of α-helix mimetics. *Org. Biomol. Chem.* **12**, 6794-6799 (2014).
